# A New Suite of Plasmid Vectors for Fluorescence-Based Imaging of Root Colonizing Pseudomonads

**DOI:** 10.3389/fpls.2017.02242

**Published:** 2018-02-01

**Authors:** Rosemarie Wilton, Angela J. Ahrendt, Shalaka Shinde, Deirdre J. Sholto-Douglas, Jessica L. Johnson, Melissa B. Brennan, Kenneth M. Kemner

**Affiliations:** ^1^Biosciences Division, Argonne National Laboratory, Argonne, IL, United States; ^2^Center for Synchrotron Radiation Research and Instrumentation, Illinois Institute of Technology, Chicago, IL, United States

**Keywords:** broad-host vector, fluorescent protein, rhizosphere, *Pseudomonas*, pVS1, plant growth promotion, biocontrol strain

## Abstract

In the terrestrial ecosystem, plant–microbe symbiotic associations are ecologically and economically important processes. To better understand these associations at structural and functional levels, different molecular and biochemical tools are applied. In this study, we have constructed a suite of vectors that incorporates several new elements into the rhizosphere stable, broad-host vector pME6031. The new vectors are useful for studies requiring multi-color tagging and visualization of plant-associated, Gram-negative bacterial strains such as *Pseudomonas* plant growth promotion and biocontrol strains. A number of genetic elements, including constitutive promoters and signal peptides that target secretion to the periplasm, have been evaluated. Several next generation fluorescent proteins, namely mTurquoise2, mNeonGreen, mRuby2, DsRed-Express2 and E2-Crimson have been incorporated into the vectors for whole cell labeling or protein tagging. Secretion of mTurquoise2 and mNeonGreen into the periplasm of *Pseudomonas fluorescens* SBW25 has also been demonstrated, providing a vehicle for tagging proteins in the periplasmic compartment. A higher copy number version of select plasmids has been produced by introduction of a previously described *repA* mutation, affording an increase in protein expression levels. The utility of these plasmids for fluorescence-based imaging is demonstrated by root colonization of *Solanum lycopersicum* seedlings by *P. fluorescens* SBW25 in a hydroponic growth system. The plasmids are stably maintained during root colonization in the absence of selective pressure for more than 2 weeks.

## Introduction

Plant growth and crop productivity are functions of nutrient resource supply and acquisition. Soil microbes play an important role in making nutrients available for plant uptake (Lugtenberg and Kamilova, 2009; Tkacz and Poole, 2015). Plants have developed a complex association with different soil microbes, with microbes forming an intimate association with the plant roots (Danhorn and Fuqua, 2007). Exchange of nutrients and carbon occurs at this interface. Amongst Pseudomonads, the collective activity of transmembrane transporters, the transportome, is predictive of the ecological niche occupied in the rhizosphere (Silby et al., 2009; Larsen et al., 2015). In a laboratory model system, it was shown that capacities of different *Pseudomonas* plant growth promotion (PGP) strains to protect plants from nutrient stress are a function of the respective bacterial transportomes (Shinde et al., 2017). Herein we describe development of an enhanced set of plasmid vectors which will be useful for future studies of the molecular mechanisms of nutrient exchange processes and in particular to observe and study the association of plants with different microbes in the rhizosphere.

Plasmid pVS1 is a 30-kb non-conjugative mobilizable plasmid isolated from *Pseudomonas aeruginosa* PAT (Stanisich et al., 1977), a clinically isolated strain that was instrumental in the early genetic analysis of *P. aeruginosa* by virtue of a second, conjugative plasmid FP2 (Holloway, 1969; Watson and Holloway, 1978). Although not maintained in *Escherichia coli*, plasmids which included the replication (*rep*) and stability (*sta*) regions of pVS1 could be established in numerous *Pseudomonas* species as well as in *Agrobacterium tumefaciens* and *Rhizobium leguminosarum* (Itoh et al., 1984; Itoh and Haas, 1985). Cloning vectors bearing the minimal pVS1 replicon have now been demonstrated to be maintained in a number of plant-associated, Gram-negative bacterial strains, even under non-selective conditions (Van den Eede et al., 1992; van der Bij et al., 1996; Heeb et al., 2000; Miller et al., 2000; Stuurman et al., 2000; Lagendijk et al., 2010). Such vectors are suitable for studying bacterial colonization in the rhizosphere or other experiments in which the use of antibiotics is undesirable. A particularly useful derivative of pVS1 is the 8.3-kb shuttle vector pME6031 (Heeb et al., 2000). This plasmid contains the minimal 3.8-kb segment of the pVS1 replicon as well as a p15A origin of replication for propagation in *Escherichia coli.* Other features include a multiple cloning site, T4 transcription terminator, and a repressible tetracycline resistance cassette from plasmid RK2. In addition, the p15A replicon contains an origin of transfer (*oriT*), enabling mobilization in the presence of the helper plasmid pRK2013 (Heeb et al., 2000).

For the present study, we have screened a series of genetic elements in the pME6031 vector backbone. These include constitutive promoters, a number of next generation fluorescent proteins for whole-cell labeling or protein tagging, and *E. coli* signal peptides to target tagged proteins to the periplasmic space, potentially through either the signal recognition particle (SRP)-dependent general secretory (Sec) or twin-arginine translocation (Tat) pathways. Plasmid variants that incorporate a previously described mutation of the pVS1 RepA protein to increase the plasmid copy number (Itoh et al., 1988), and by extension protein expression levels, have also been produced. In addition, minor restriction site modifications were introduced to facilitate cloning. The full pME6031 vector and several of the derivatives reported herein were confirmed using next generation sequencing (NGS) methods, and minor variations from earlier published sequences are described. We have examined fluorescent protein expression and plasmid stability in several *Pseudomonas* plant growth promoting or biocontrol strains. A simple model system to monitor root colonization of a PGP strain, *P. fluorescens* SBW25, on tomato seedlings confirms the use of these plasmids as stable elements for the investigation of plant–microbe interactions.

## Materials and Methods

### Materials

Restriction and DNA modifying enzymes were from New England Biolabs (Ipswich, MA, United States). KOD polymerase (EMD Millipore, Billerica MA, United States) was used for PCR amplification. Plasmid miniprep and PCR purification kits were from QIAGEN (Germantown, MD, United States). Synthetic DNA and oligonucleotides were obtained from Integrated DNA Technologies (IDT; Coralville, IA, United States). Unless otherwise indicated, all other chemicals and reagents were from Sigma (St. Louis, MO, United States) or Thermo Fisher Scientific (Waltham, MA, United States).

### Bacterial Strains, Plasmids and Culture Conditions

*Pseudomonas fluorescens* SBW25 (Rainey and Bailey, 1996) was a generous gift from Dr. Gail Preston, Department of Plant Sciences at the University of Oxford, Oxford, United Kingdom. *P. protegens* Pf-5 (Howell and Stipanovic, 1979; Howell, 1980) was from ATCC (BAA-477). *P. fluorescens* Pf0-1 (Compeau et al., 1988) was kindly provided by Dr. Mark Silby, University of Massachusetts Dartmouth, North Dartmouth, MA. *P. fluorescens* WH6 (Banowetz et al., 2008) was a kind gift of Dr. Mark Azevedo, USDA-ARS Forage Seed and Cereal Research Center, Corvallis OR. *P. protegens* CHA0 (Stutz et al., 1986) was obtained from the Culture Collection of Switzerland (CCOS; collection number CCOS 2^T^). Plasmids pME6031, pME6012 and pME6032 were obtained from CCOS (part numbers CCOS855, CCOS854 and CCOS856, respectively). Plasmids were maintained in *E. coli* strain DH5α, and all cloning and mutagenesis steps were propagated in the same strain.

Chemically competent *E. coli* was prepared with the *Mix* & *Go E. coli* Transformation Kit (Zymo Research, Irvine, CA, United States). *E. coli* was cultured at 37°C in LB medium (Sambrook et al., 1989) containing 10 μg/ml tetracycline. *Pseudomonas* strains were transformed by electroporation (Wang et al., 2009) using 10–500 ng plasmid DNA. Transformed cells were selected by plating on LB-agar containing 80 μg/ml tetracycline, and grown at 30°C for 2 days to develop colonies. For liquid cultures, *Pseudomonas* strains were grown in LB medium at 25°C, and, when used, tetracycline was added at a final concentration of 80 μg/ml.

### Mutagenesis of the pME6031 Vector Backbone

Site directed mutagenesis was performed using the QuickChange Lightning Site-Directed Mutagenesis or Multi Site-Directed Mutagenesis Kits (Agilent Technologies, Santa Clara, CA, United States). Mutations were verified by Sanger sequence analysis (University of Chicago Comprehensive Cancer Center DNA Sequencing & Genotyping Facility, Chicago, IL, United States). Full plasmids were sequenced using next-generation (NGS) methods (CCIB DNA Core Facility; Massachusetts General Hospital, Cambridge, MA, United States). Mutagenic primers are listed in Supplementary Table S1 and all sequencing primers used in the present study are listed in Supplementary Table S2.

### Design of DNA Constructs Coding for Fluorescent Proteins, Promoters and Other Elements

Amino acid sequences for the monomeric fluorescent proteins mTurquoise2, mNeonGreen, and mRuby2, were obtained from published sources (Goedhart et al., 2012; Shaner et al., 2013; Bajar et al., 2016). The sequences were reverse translated, codon optimized for expression, and energy minimized to reduce RNA secondary structure using the Codon/Expression Optimization tool (Blue Heron Biotechnology, Inc.^1^). The *P. fluorescens* [gbbct] codon usage table was selected for codon optimization. Amino acids at the N-terminus of monomeric fluorescent proteins were modified to introduce an EcoRI site. The tetrameric red and far-red fluorescent proteins, DsRed-Express2 and E2-Crimson (Strack et al., 2008, 2009), were amplified directly from plasmids kindly provided by Dr. Benjamin S. Glick, Department of Molecular Genetics and Cell Biology, University of Chicago, Chicago, IL, United States. Signal peptides of the *E. coli* K-12 TorT (AAC74079.1) and TorA (NP_415517.1) proteins were identified using PRED-TAT^2^ (Bagos et al., 2010), and DNA coding sequences for the peptides were used without modification.

The DNA sequence of the *Amaranthus hybridus P*_psbA_ promoter (Wang et al., 2007) was kindly provided by Dr. Kirankumar Mysore, Plant Biology Division, The Samuel Roberts Noble Foundation, Ardmore, OK, United States. The sequence was used as provided, except for removal of a HindIII site. The *P_c_* promoter sequence was derived from the class III integron of *Delftia acidovorans* C17 (Xu et al., 2013). A NcoI site was removed. The ribosome binding site (RBS) was derived from gene *10* of bacteriophage T7 (Olins et al., 1988); the XbaI site upstream of the RBS was removed. To ensure that elimination of restriction sites in the vicinity of promoters or RBS did not alter expression levels, fluorescent protein expression was first characterized with promoters that retained those sites. Expression levels were unaffected by removal of the aforementioned restriction sites (data not shown).

### Construction of Fluorescent Protein Expression Vectors

Synthetic DNA (gBlocks Gene Fragments) encoding various combinations of promoters, signal peptides, monomeric fluorescent proteins and C-terminal hexahistidine tags (His-tags) was obtained from Integrated DNA Technologies (IDT; Coralville, IA, United States). In the current constructs, His-tags, where present, are isolated from coding sequences by a stop codon. Relevant segments of gBlocks (see Supplementary Table S3 for sequences) were amplified by PCR using primers that incorporated appropriate restriction sites. Sequences of PCR primers are listed in Supplementary Table S4. PCR products were cloned into pME6031 derivatives by restriction enzyme digestion and ligation. DsRed-Express2 and E2-Crimson coding sequences were amplified directly from plasmid DNA. Using these methods, a series of plasmids expressing various fluorescent proteins driven by *P_c_* or *P*_psbA_ promoters was constructed. In some plasmids, fluorescent protein coding sequences were appended to coding sequences for TorT or TorA signal peptides. All inserted sequences were verified by Sanger sequencing (see Supplementary Table S2 for sequencing primers), and select plasmids were submitted for NGS analysis.

### Plasmid Stability in *Pseudomonas* Strains

*Pseudomonas* strains containing plasmids of interest were grown overnight in 15 mL LB medium containing 80 μg/mL tetracycline at 25°C, 250 rpm. The next day, cultures were diluted 10^6^-fold into 50 mL LB (no antibiotic) and grown for 24 h. This was repeated two more times (approximately 60 generations total). After the third day, an aliquot of each culture was removed and diluted 10^5^-fold, plated on LB agar and grown overnight at 30°C. The following day, 100 colonies were patch-plated onto both LB agar and LB agar plates containing 80 μg/mL tetracycline. The number of colonies retaining growth on selective medium were counted and presented as a percentage of the total colonies patch-plated. The effect of *Pseudomonas* strain on stability was assessed by non-parametric Oneway analysis using Wilcoxon/Kruskal–Wallis Tests (Rank Sums). Effect of different engineered plasmids on stability in *P. fluorescens* SBW25 was performed using analyses of variance (ANOVA) followed by Tukey’s HSD to highlight plasmid response means that differed between wild type (pME6031) and other engineered plasmids in *P. fluorescens* SBW25.

### Mini-Tn7 Transposon Strains

Mini-Tn7 transposon vector pUC18T-mini-Tn7T-Gm (accession no. AY599232) and helper plasmid pTNS3 (accession no. EU215432) (Choi et al., 2005; Choi and Schweizer, 2006) were a generous gift of Dr. Herbert Schweizer, University of Florida, Gainesville, FL, United States. Plasmid pTNS3 was maintained in strain PIR1 (*pir*-116; ThermoFisher). *P_c_*-fluorescent protein-T4 terminator segments were amplified by PCR from pSW002-based plasmids and inserted into the mini-Tn7 vector using GeneArt Seamless Cloning and Assembly (ThermoFisher) and verified by sequence analysis. Mini-Tn7 plasmids containing fluorescent protein expression elements were co-electroporated into *P. fluorescens* SBW25 with pTNS3 (100 ng each plasmid) and plated on LB-agar containing 15 μg/ml gentamycin. Several of the resulting colonies were then re-streaked on gentamycin plates, and single colonies were selected for further analysis. Insertion was confirmed by PCR amplification of genomic DNA using primers designed for the *P. fluorescens* SBW25 *glmS* insertion site. PCR products were sequenced to verify the sequence of the *P_c_*-fluorescent protein element. Double-labeled *P. fluorescens* SBW25 strains were obtained by electroporation of appropriate pSW002-based plasmids into mini-Tn7 strains expressing mNeonGreen or DsRed-Express2.

### Evaluation of mNeonGreen Expression

To evaluate expression levels of mNeonGreen driven from different pME6031 derivatives, promoters and *P. fluorescens* strains, overnight cultures were diluted in fresh LB medium containing appropriate antibiotics to an OD_600__nm_ of 0.05. Two hundred microliters of diluted culture was transferred to sterile, covered, black microwell plates with clear bottoms (Corning 3603). The microplates were incubated at 30°C with continuous shaking at 120 rpm in a Hidex Sense microplate reader (LabLogic Systems, Inc., Brandon, FL, United States). Culture absorbance (OD_600__nm_) and fluorescence (Ex 485/10 nm; Em 535/20 nm) measurements were taken at 15 min intervals over a period of 24 h. Two independent cultures were started for each plasmid/strain combination, and three replicate wells were analyzed for each culture. The normalized fluorescence was determined at the start of stationary phase by dividing the fluorescence value by the culture optical density. Data analysis and graphing were performed using GraphPad Prism Version 7 for Windows, GraphPad Software, La Jolla, CA, United States, www.graphpad.com. The mean and standard deviation for each data set is presented in the histograms.

### Colonization of Bacteria on Tomato Roots in a Hydroponic System

Tomato seeds (*Solanum lycopersicum*) were surface sterilized by washing once in 0.4% (v/v) Tween 20 followed by 3 min in a 5% dilution of household bleach and six rinses with sterile water. Seeds were imbibed in water in the dark overnight and then transferred to sterile glass Petri dishes containing water-saturated Kim-Wipes. The dishes were stored in the dark at 4°C overnight and then transferred to a plant growth chamber for germination, under a 14 h/10 h light/dark cycle, a day/night temperature regime of 25/20°C, photosynthetically active radiation (PAR) of 200 μmol m^-2^ s^-1^, and constant relative humidity of 70%. When the first true leaves emerged, the seedlings were transferred to 50 ml conical centrifuge tubes filled with sterile nutrient solution, Desai et al. (2014) described in Supplementary Table S5. The seedlings were held in place with collars constructed from sterile foam plugs, and tubes were wrapped with aluminum foil to eliminate light from the root zone (Supplementary Figure S1). Transplanted seedlings were acclimated for 7–14 days prior to inoculation. For inoculation, overnight cultures of bacterial strains of interest were washed once in nutrient solution and resuspended in the same solution at a final OD_600__nm_ of 2.0. Each seedling was inoculated with 3 ml of washed cell suspension. The final concentration of bacterial cells was estimated to be 5 × 10^8^ cfu/ml. Two seedlings were inoculated for each plasmid/strain tested. Nutrient solution was aseptically replenished as needed during the incubation period. At 1-week intervals, two lateral roots (ranging from 2 to 6 cm in length) were excised from each plant and rinsed in nutrient solution. Root segments (1 cm) were placed on microscope slides in a drop of 50% glycerol. All manipulations of seedlings were conducted in a laminar flow hood using aseptic techniques. Cross contamination of seedlings with fluorescent strains was not observed as verified by examination of uninoculated control plants.

### Fluorescence Microscopy and Confocal Microscopy

To image bacterial cells, cultures were grown overnight in LB containing 80 μg/mL tetracycline, washed once in nutrient solution and resuspended in the same medium. Small (3 μl) volumes of cells were mounted on microscope slides. Inoculated seedling root sections were prepared as described above. Microscopy was performed on two separate microscopes. On the first setup a Nikon Eclipse Ti-E inverted microscope was equipped with a Crest X-Light spinning disk confocal system (Crestoptics, Rome, Italy), SPECTRA X and LIDA light engines (Lumencor, Inc., Beaverton, OR, United States), a QuantEM:512SC electron-multiplying CCD camera (Photometrics, Tucson, AZ, United States), and a Nikon CFI60 Plan Apochromatλ 100x oil immersion objective (N.A. 1.45, W.D. 0.13 mm) or a Nikon CFI160 Plan Fluor (N.A. 0.3, W.D. 16 mm). On the second setup an inverted Nikon C2+ laser-scanning confocal microscope was used (Nikon, Melville, NY, United States). An Eclipse Ti-E inverted microscope equipped with perfect focus system, an automated stage, and with 10x, 20x, and 100x objective lenses (CFI Plan Fluor 10X, NA 0.3, WD 16 mm; CFI Plan Apochromat Lambda 20x, NA 0.75, WD 1.00 mm; CFI Plan Apo Lambda 100x, NA 1.5, WD 130 um, respectively) was used for single image, time series, and z-stack acquisitions. Laser illumination emission at 488 nm coupled with a 525/50-nm excitation filter was used to capture mNeonGreen fluorescence, and laser illumination of 561 nm coupled with a 595/50-nm excitation filter was used to capture DsRed fluorescence. All images were prepared with the Fiji distribution of ImageJ (Schindelin et al., 2012).

## Results

### Modification of Plasmid pME6031 and Sequence Analysis

**FIGURE 1:**
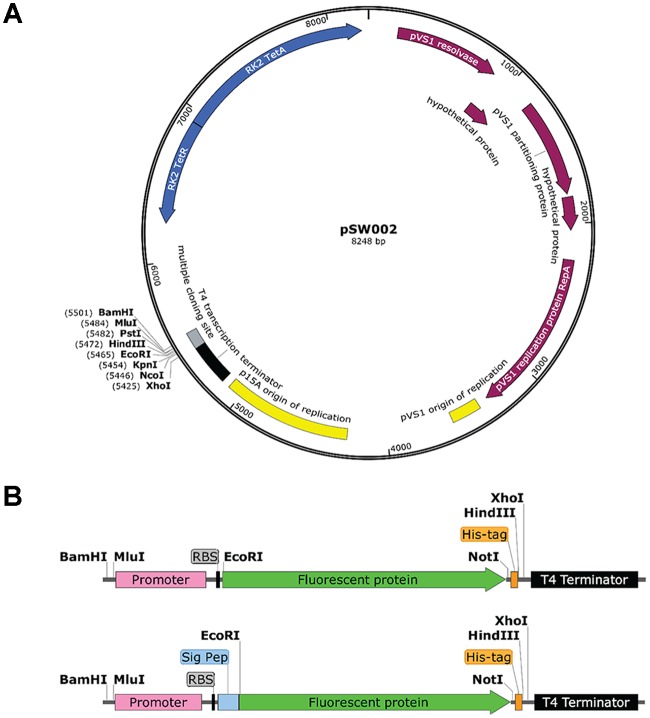
**(A)** Schematic drawing of the rhizosphere-stable expression vector pSW002 (derived from pME6031 following removal of selected restriction sites). **(B)** Detail of the arrangement of promoter, protein elements and available restriction sites after insertion into the BamHI and XhoI sites of pSW002. The lower segment illustrates the arrangement of constructs containing signal peptides for secretion of fluorescent protein into the periplasmic space. The His-tag can be incorporated into coding segments by site-directed mutagenesis or by using cloning procedures that remove the stop codon from the C-terminal end of the fluorescent protein. The diagrams were drawn with SnapGene^®^ software (GSL Biotech; snapgene.com).

Mutations of the pVS1 replication/stability region have been shown to modulate plasmid copy number (Itoh et al., 1988). In particular, the Ala-246 to Val (A246V) mutation of RepA doubles the copy number of this family of plasmids from approximately 6–13 copies per cell (Heeb et al., 2000). Site-directed mutagenesis was used to construct a variant of pME6031 that contains the A246V mutation as a means to enhance the expression of fluorescent proteins or other protein constructs. The resulting plasmid was named pSW001. Secondly, in order to simplify future cloning tasks, site-directed mutagenesis was used to remove several restriction sites from pME6031. These include NotI(844), NotI(2377), NotI(3667), XbaI(8291) and BamHI(1) (the location on pME6031 is given in parentheses and is numbered according to published pME6031 sequence, accession number AF118811). NotI(844) and NotI(2377) reside in coding regions of the pVS1 resolvase and pVS1 replication protein, respectively, so silent mutations were used to preserve the amino acid sequences. The resulting plasmid was named pSW002. A third variant (pSW003) combined the RepA and restriction site mutations. The three plasmids were fully sequenced by next-generation sequencing (NGS) methods to verify that no spurious mutations had been introduced by the mutagenesis procedures. The parental plasmid pME6031 was fully sequenced as well. Several minor discrepancies between the published sequence of pME6031 (accession number AF118811) and the NGS data are listed in **Table 1**. Most notably, a 66 bp deletion of a GC rich region is observed between the RK2 tetracycline resistance gene and the multiple cloning site. We performed NGS analysis of the related plasmids pME6012 and pME6032 (Heeb et al., 2000) and uncovered the same deletion, suggesting that the deletion occurred earlier in the lineage of these plasmids and was propagated in the derivatives. The absence of this GC-rich region, which is devoid of features, is not expected to influence the function of the plasmid. The deletion was also confirmed by standard Sanger sequencing methods. Another, potentially more relevant, mutation was a single base deletion that removes a reported BamHI site. This was also revealed during site-directed mutagenesis experiments and was confirmed by NGS. For reference, the NGS data for pME6031 is provided in the Supplementary Data. The pSW002 plasmid map is illustrated in **Figure 1A**.

**Table 1:** Sequence variations of plasmid pME6031 confirmed by Next Generation Sequencing.

Location	Observation	Comment
1	Deletion of G	Removes a BamHI site
3802	Deletion of G	Between pVS1 ori and p15A ori
5137	Deletion of C	Near T4 transcription terminator
5526(CAGC)5527	Insertion of CAGC	Adjacent to the MCS
6079(del)6146	Deletion	66 base deletion; GC-rich region
8286(GG)8287	Insertion of GG	Adjacent to XbaI site

*Numbering is according to Accession number AF118811.*

### Stability of pME6031 and Variants *in Vitro* under Non-selective Growth Conditions

Previous studies have shown that pME6031 and other pVS1-based plasmids are retained in Pseudomonads for many generations under non-selective conditions. This includes strains such as *P. protegens* CHA0 (Heeb et al., 2000), *P. fluorescens* WCS365 (Bloemberg et al., 2000) and *P. putida* PCL1445 (Lagendijk et al., 2010). Similarly, plasmid pME290, which contains the same segment of the pVS1 replicon as pME6031, is stable in a number of pseudomonad hosts (Itoh and Haas, 1985). We tested pME6031 maintenance in several *Pseudomonas* plant growth promoting strains under study in our laboratory, particularly *P. fluorescens* SBW25, *P. fluorescens* Pf0-1, *P. fluorescens* WH6 and *P. protegens* Pf-5. As a control, we included *P. protegens* CHA0, a strain in which pME6031 has been shown to highly stable (Heeb et al., 2000). We performed liquid culture experiments, passaging the strains under non-selective conditions. After 60 generations the cultures were plated, and the resulting colonies were patch-plated onto non-selective and selective plates. Consistent with earlier reports, pME6031 was 100% retained in *P. protegens* CHA0 (**Table 2**). The maintenance of pME6031 in *P. fluorescens* SBW25, *P. fluorescens* Pf0-1 and *P. protegens* Pf-5 ranged from 89% to 97%; these are statistically different from each other (ChiSquare 11.5 and *p*-value is 0.0215^∗^). The plasmid was somewhat less stable without selection in *P. fluorescens* WH6, with only 28% of patched colonies retaining the plasmid. In order to confirm that restriction site knock-outs in the pVS1 replicon did not impact plasmid maintenance, and also to assess the impact of the high copy number RepA mutation, A246V, we tested maintenance of pSW001, pSW002 and pSW003 in *P. fluorescens* SBW25. The plasmid pSW002 exhibited a level of stability (92%) similar to pME6031, confirming that the restriction site mutations did not impact plasmid replication or segregation under these conditions. Predictably, the higher copy number plasmids, pSW001 and pSW003, exhibited improved stability and were maintained in nearly 100% of patch-plated colonies. Plasmid stability significantly varies (F-ratio 100 and *p*-value < 0.0001^∗^). Although most strains exhibited at least some plasmid loss during passage in liquid culture, it is well recognized that such experiments can artificially amplify the apparent rate of plasmid loss due to higher growth rate of plasmid-free cells (Boe, 1996; Boe and Rasmussen, 1996; Olsson et al., 2004; Lau et al., 2013). Stability of plasmids expressing fluorescent proteins and during root colonization was assessed in experiments described below.

**Table 2:** Maintenance of pME6031 and variants after 60 generations under non-selective liquid culture conditions.

Stability of pME6031 in different *Pseudomonas* strains^b^			
**Strain**	***n*^a^**	**Percent resistant colonies**	

*P. fluorescens* SBW25	4	89% (±0.01)	
*P. fluorescens* Pf0-1	2	95% (±0.01)	
*P. fluorescens* WH6	3	28% (±0.1)	
*P. protegens* CHA0	2	100% (±0)	
*P. protegens* Pf-5	2	97% (±0.01)	

**Stability of engineered plasmid variants in *P. fluorescens* SBW25^c^**			

**Plasmid**	***n***	**Percent resistant colonies**	**Tukey HSD**

pME6031	4	89% (±0.01)	B
pSW001	3	100% (±0)	A
pSW002	2	92% (±0.01)	B
pSW003	2	100% (±0.01)	A

^a^*Number of individual stability trials conducted. ^*b*^Oneway analysis using Wilcoxon / Kruskal-Wallis Tests (Rank Sums) shows statistically significant difference in plasmid stability between the strains (ChiSquare 11.5028 and p-value 0.0215^∗^). ^*c*^Stability of engineered plasmid variants in *P. fluorescens* SBW25 are significantly different (F ratio 100.01 and *p*-value < 0.0001^∗^). Plasmids not connected by the same identifying letter are significantly different.*

### Construction of Vectors for Constitutive Fluorescent Protein Expression

Synthetic DNA constructs consisting of constitutive promoters and the bacteriophage T7 gene *10* ribosome binding site were inserted into the vectors pSW002 or pSW003 as MluI/EcoRI fragments. Two different promoters were assessed, a *P_c_* promoter from a class 3 integron (Xu et al., 2013) and a chloroplast promoter, *P*_psbA_ (Wang et al., 2007). Coding sequences for the monomeric fluorescent proteins mNeonGreen (Shaner et al., 2013), mTurquoise2 (Goedhart et al., 2012) and mRuby2 (Shaner et al., 2008) were codon optimized for expression in *Pseudomonas fluorescens*, and inserted as EcoRI/XhoI fragments between *P_c_* or *P*_psbA_ and the T4 transcription terminator on pME6031. A sequence coding for a C-terminal hexahistidine affinity tag was also added to vectors containing the monomeric fluorescent proteins at this stage. At present, expression of the affinity tag is blocked by a stop codon at the end of the fluorescent protein sequence. However, the affinity tag can be easily incorporated, if desired, by removal of the stop codon using site-directed mutagenesis or by cloning into the NotI site that precedes the tag coding sequence. Coding sequences for the tetrameric DsRed-Express2 and E2-Crimson (Strack et al., 2008, 2009) were cloned from existing plasmids as described under *Materials and Methods*. Details of the cloning site architecture are illustrated in **Figure 1B** and all plasmids constructed for the present study are listed in **Table 3**. Properties of fluorescent proteins available in the suite of plasmids are listed in **Table 4**. As demonstrated below, this set of vectors will facilitate tagging and visualization of bacterial root colonization across a wide spectral range. Additionally, vectors containing monomeric proteins have potential use for construction of fusion tags for protein localization studies.

**Table 3:** List of new vectors created from pME6031.

Plasmid name	Relevant characteristics
pSW001	pME6031 with high copy number mutation
pSW002	pME6031 with restriction sites^1^ removed
pSW003	pME6031 with high copy number mutation^2^ + restriction sites^1^ removed
pSW002-*P*_c_	Empty vector + *P*_c_ promoter
pSW002-*P*_c_-mTurquoise2	*P*_c_ promoter + cytosolic expression of mTurquoise2
pSW002-*P*_c_-mNeonGreen	*P*_c_ promoter + cytosolic expression of mNeonGreen
pSW002-*P*_c_-mRuby2	*P*_c_ promoter + cytosolic expression of mRuby2
pSW002-*P*_c_-DsRed-Express2	*P*_c_ promoter + cytosolic expression of DsRed-Express2
pSW002-*P*_c_-E2-Crimson	*P*_c_ promoter + cytosolic expression of E2-Crimson
pSW002-*P*_c_-TorA	Empty vector + *P*_c_ promoter + TorA signal peptide
pSW002-*P*_c_-TorA-mTurquoise2	*P*_c_ promoter + periplasmic targeting of mTurquoise2
pSW002-*P*_c_-TorA-mNeonGreen	*P*_c_ promoter + periplasmic targeting of mNeonGreen
pSW002-*P*_c_-TorT	Empty vector + *P*_c_ promoter + TorT signal peptide
pSW002-*P*_c_-TorT-mTurquoise2	*P*_c_ promoter + periplasmic targeting of mTurquoise2
pSW002-*P*_c_-TorT-mNeonGreen	*P*_c_ promoter + periplasmic targeting of mNeonGreen
pSW002-*P*_psbA_	Empty vector + *psbA* promoter
pSW002-*P*_psbA_-mTurquoise2	*psbA* promoter + cytosolic expression of mTurquoise2
pSW002-*P*_psbA_-mNeonGreen	*psbA* promoter + cytosolic expression of mNeonGreen
pSW002-*P*_psbA_-DsRed-Express2	*psbA* promoter + cytosolic expression of DsRed-Express2
pSW002-*P*_psbA_-E2-Crimson	*psbA* promoter + cytosolic expression of E2-Crimson
pSW003-*P*_c_-mNeonGreen	High copy vector + *P*_c_ promoter + cytosolic expression of mNeonGreen
pSW003-*P*_psbA_-mNeonGreen	High copy vector + *psbA* promoter + cytosolic expression of mNeonGreen

*The format of each plasmid name is: base plasmid-promoter-periplasmic signal sequence (if present)-fluorescent protein. ^*1*^NotI(844), NotI(2377), NotI(3667), XbaI(8291) and BamHI(1). ^*2*^RepA(A246V).*

**Table 4:** Properties of fluorescent proteins used in this study.

Protein	λ_ex_ (nm)	λ_em_ (nm)	𝜀 (mM^-1^ cm^-1^)^a^	ϕ^b^	Brightness^c^	Reference
mTurquoise2	434	474	30	0.93	27.9	Goedhart et al., 2012
mNeonGreen	506	517	116	0.80	92.8	Shaner et al., 2013
mRuby2	559	600	113	0.38	43	Lam et al., 2012
DsRed-Express2	554	591	35.6	0.42	15.0	Strack et al., 2008
E2-Crimson	611	646	126	0.23	29	Strack et al., 2009

^a^*Extinction coefficient. ^b^Quantum yield. ^c^Brightness is the product of the extinction coefficient and quantum yield.*

### Evaluation of Constitutive Promoters

**FIGURE 2:**
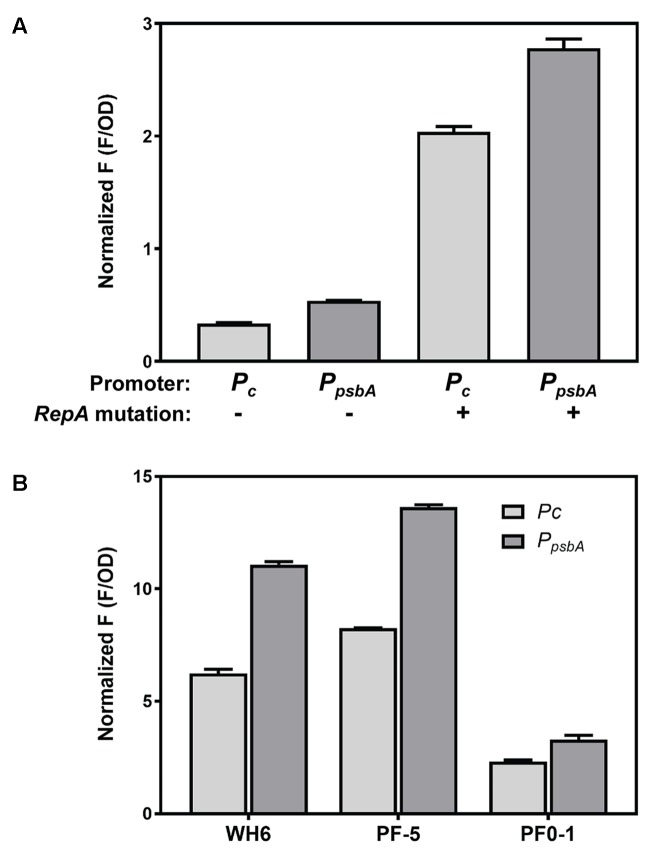
Expression of mNeonGreen from *P*_c_ and *P*_psbA_ promoters in high and low copy plasmids and in different *Pseudomonas* strains. **(A)** Comparison of mNeonGreen expression in *P. fluorescens* SBW25 from *P*_c_ and *P*_psbA_ and effect of RepA A246V mutation to increase plasmid copy number. **(B)** Effect of different promoters on mNeonGreen expression in *P. fluorescens* WH6, *P. protegens* Pf-5 and *P. fluorescens* Pf0-1. Each histogram bar depicts the mean and standard deviation of the normalized fluorescence generated from two independent starter cultures. Three replicate wells were averaged for each culture. Fluorescence values at early stationary phase were normalized according to culture optical density at 600 nm. In order to optimize fluorescence measurements, experiments A and B were performed using different plate reader gain settings; therefore normalized F measurements should not be compared between the two graphs.

The effect of promoter, plasmid copy number and *Pseudomonas* host strain on expression level was evaluated for mNeonGreen (**Figure 2A**). We anticipated that the two promoters would provide different levels of expression in *Pseudomonas* host strains, possibly allowing some regulation of expression level based on the chosen promoter. Although the *P*_psbA_ promoter showed obviously stronger constitutive expression than *P*_c_ in the *E. coli* cloning strain (data not shown), differences in expression levels driven by *P*_c_ and *P*_psbA_ promoters in all *Pseudomonas* strains varied by less than twofold (**Figures 2A,B**). A more significant increase in expression was observed with the higher copy number plasmid, pSW003. Earlier studies indicated that the A246V RepA mutation roughly doubled pVS1 derivative plasmids, from about 6–13 copies per cell (Heeb et al., 2000). In *P. fluorescens* SBW25, incorporation of the A246V mutation led to a 5 to 6-fold increase in mNeonGreen expression. (**Figure 2A**).

During analysis of the *P*_psbA_ nucleotide sequence, we noticed it contained a translation start site for the protein encoded by *psbA*, the D1 protein of the photosystem II reaction center. The region contains a cryptic RBS (Kim and Mullet, 1994), and it is not clear whether a D1 peptide is expressed in Pseudomonads. We constructed two variants of *P*_psbA_ that deleted this region, Del1 removed the D1 coding sequence, and Del2 deleted D1 and extended through the cryptic RBS. Neither of the deletions appreciably affected the expression of mNeonGreen in *P. fluorescens* SBW25 (Supplementary Figure S2A), so the promoter deletions were not studied further. We also tested expression of mNeonGreen from *P_tac_* in *P. fluorescens* SBW25. Because *Pseudomonas* strains do not contain a *lac* repressor, the *E. coli lac* promoter and derivatives act as constitutive promoters. Although *P_tac_* produced normalized expression levels similar to *P*_c_ and *P*_psbA_, the bacterial growth rate was reduced (Supplementary Figures S2A,B), so this promoter was not used further.

### Export of Fluorescent Proteins to the Periplasmic Space of *P. fluorescens* SBW25

**FIGURE 3:**
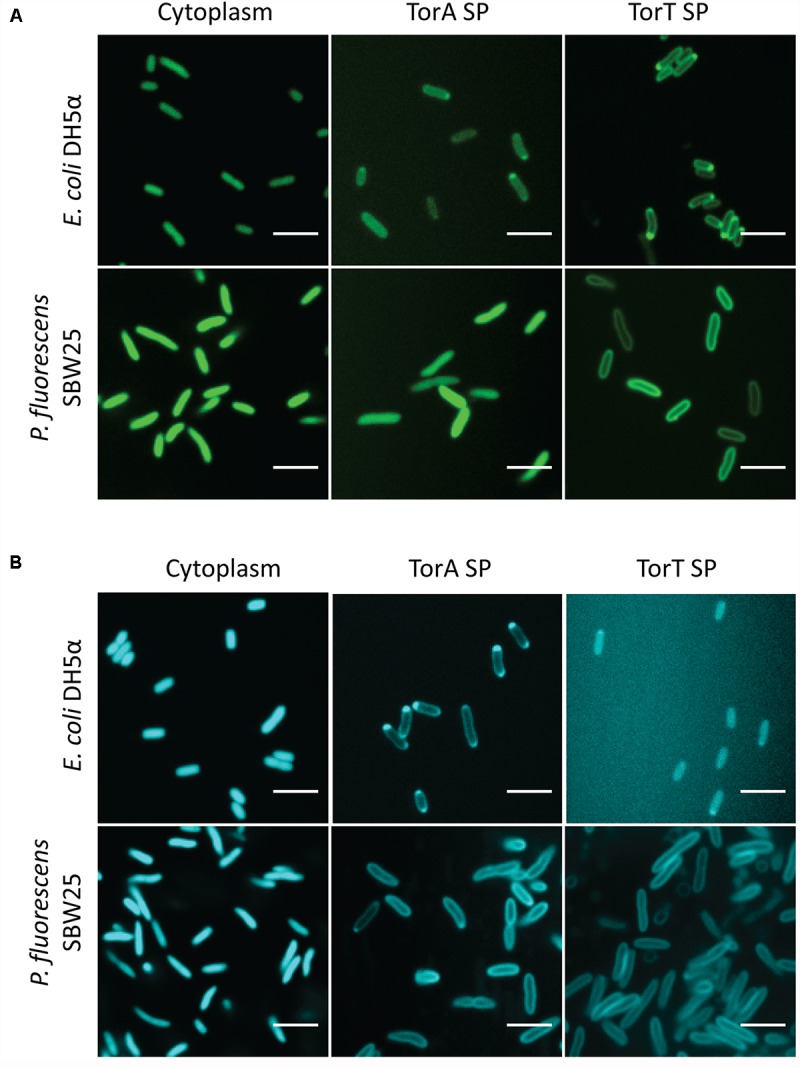
Confocal images of **(A)** mNeonGreen and **(B)** mTurquoise2 expressed in the cytoplasm and periplasm of *E. coli* DH5α and *P. fluorescens* SBW25. Each montage compares the localization of the fluorescent protein in the two strains in the absence of a signal peptide (column 1) or driven by the TorA or TorT signal peptide (SP; columns 2 and 3).

Monomeric fluorescent proteins mTurquoise2 and mNeonGreen were evaluated for secretion in *P. fluorescens* SBW25. Export of these fluorescent proteins to the periplasmic space is of interest for tagging and localization of fusion proteins and for potential use in FRET applications. In the present study we assessed secretion using signal peptides from two *E. coli* proteins, TorT and TorA, which in *E. coli* are secreted through the SRP-dependent SEC and Tat pathways, respectively. The signal peptides were fused to fluorescent proteins expressed under control of *P*_c_ in pSW002, and targeting to the periplasm was evaluated by confocal fluorescence microscopy (**Figure 3**). The results indicate that mNeonGreen and mTurquoise2 secretion occurs with different signal peptide preferences. Effective secretion of mNeonGreen was observed only with the TorT signal peptide. When fused to the TorA peptide, mNeonGreen was mostly retained in the cytoplasm (**Figure 3A**). mTurquoise2 could be secreted with either the TorA or the TorT signal peptide. However, secretion with TorT yielded a very faint signal, while TorA produced bright labeling of the periplasm (**Figure 3B**). Orthogonal views in Supplementary Figure S3 confirm that mNeonGreen and mTurquoise2 are confined to the periphery of *P. fluorescens* SBW25 when exported via the preferred secretion pathways. For comparison, we tested localization of these proteins in *E. coli* DH5α, using the same expression vectors (**Figure 3**). We observed that the same signal peptide/fluorescent protein combinations were favored in *E. coli*, consistent with export through the same secretion pathways in both host strains. Although confirmation of export via *P. fluorescens* SBW25 SRP-dependent SEC or Tat pathways will necessitate additional genetic studies, these two signal peptides are nonetheless useful for empirical selection of appropriate secretion partners.

### Stability of Plasmids during Colonization of Tomato Root by *P. fluorescens* SBW25

**FIGURE 4:**
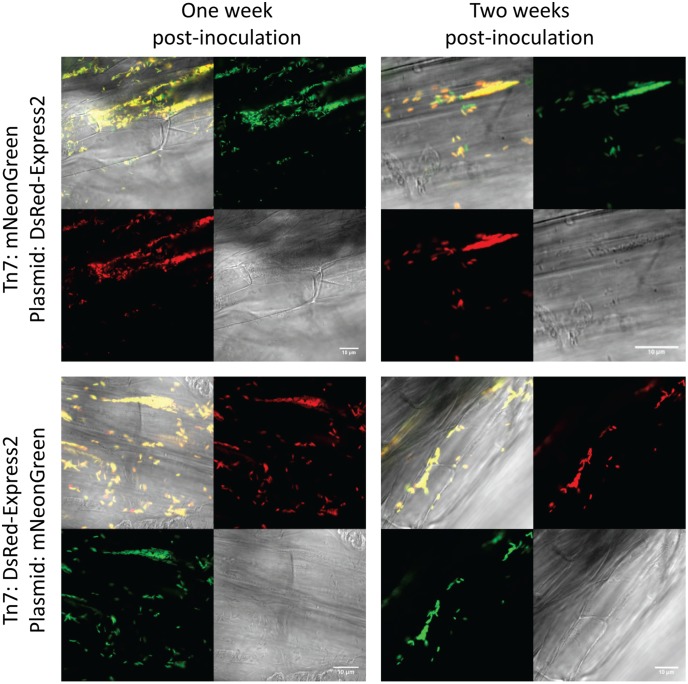
Confocal and brightfield images of tomato roots colonized with *P. fluorescens* SBW25 double labeled with mini-Tn7 transposon and plasmid. The plasmid used here is pSW002 and protein expression is driven by the *P*_c_ promoter. The fluorescent protein expressed by each genetic component is indicated at the left. In each montage, the lower right is the brightfield image, the lower left is the DsRed-Express2 image, the upper right is the mNeonGreen image, and the upper left is the overlay of all three channels. Representative colonized root sections are shown one and 2 weeks post-inoculation.

**FIGURE 5:**
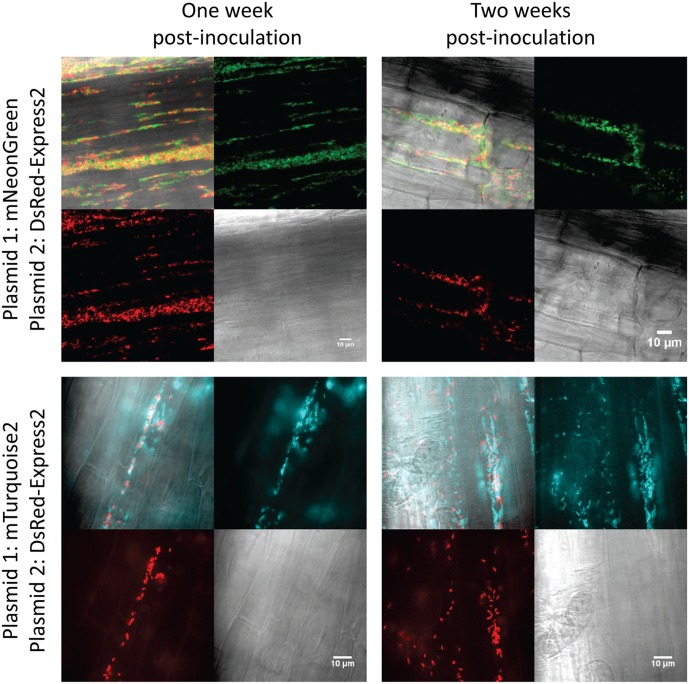
Confocal and brightfield images of tomato roots colonized with mixed populations of *P. fluorescens* SBW25 labeled with DsRed-Express2 and mNeonGreen **(top)** or DsRed-Express2 and mTurquoise2 **(bottom)**. In all cases, protein is expressed on plasmid pSW002 with expression driven by the *P*_c_ promoter. In each montage, the lower right is the brightfield image, the lower left is the DsRed-Express2 image, the upper right is the mNeonGreen **(top)** or mTurquoise2 **(bottom)** image, and the upper left is the overlay of all three channels. Representative colonized root sections are shown one and 2 weeks post-inoculation.

To test stability of the plasmids during root colonization of *P. fluorescens* SBW25, we constructed strains carrying a genome-integrated copy of mNeonGreen or DsRed-Express2. Integration was accomplished using the mini-Tn7 transposon system (Choi et al., 2005; Choi and Schweizer, 2006). The appropriate pSW002 plasmid was then introduced into each strain, generating transformants that expressed both mNeonGreen and DsRed-Express2. Expression of fluorescent protein was driven by *P*_c_ in both the plasmid and mini-Tn7 cassettes. Two-week old tomato seedlings were inoculated with the double-labeled bacteria and were grown using a simple hydroponic technique (Supplementary Figure S1). The growth medium consisted of a defined mixture of macro and micronutrients (Desai et al., 2014), and all carbon for microbial growth was derived from root exudates or other rhizodeposits (Dennis et al., 2010). Roots were excised at one and 2 week intervals following inoculation and were evaluated by confocal microscopy (**Figure 4**). A few cells which have lost plasmid are observed, however, we did not detect extensive colonization or competitive growth of the *plasmid*^–^ cells. The results indicate that the plasmids are satisfactorily maintained during root colonization in *P. fluorescens* SBW25 and should be useful for most imaging purposes. We also tested colonization of tomato roots with mixed populations of *P. fluorescens* SBW25 expressing mNeonGreen and DsRed-Express2 or mTurquoise2 and DsRed-Express2 (**Figure 5**). In both cases, comparable levels of colonization were observed for either component of the population, indicating that for these pairings, none of the plasmids produced a selective growth advantage or disadvantage. As has been described previously for *Pseudomonas* bacteria, root colonization was patchy, with heavily colonized areas interspersed with large bacteria-free zones (Chin-A-Woeng and Lugtenberg, 2004). Colonization by *P. fluorescens* SBW25 occurred primarily in the crevices between the rhizodermal cells, giving the root surface a striated appearance (Supplementary Figure S4). Furthermore, colonization was largely confined to primary and lateral roots and was not observed on the root tip or on root hairs. Additional images of tomato roots colonized by the control strain *P. protegens* CHA0 (labeled with mNeonGreen) and *P. fluorescens* SBW25 (labeled individually with all monomeric fluorescent proteins and with the high copy number vector pSW003) are provided in the supplemental data (Supplementary Figures S4–S7).

## Discussion

We have constructed a series of plasmid vectors that will be useful for a wide variety of studies requiring tagging and visualization of plant-associated, Gram-negative strains. These vectors are based on the pVS1-p15A shuttle vector, pME6031 (Heeb et al., 2000). In addition to the utility of pME6031 based vectors in *Pseudomonas* spp., vectors containing the pVS1 origin of replication have been found to be stable in a wide range of Gram-negative hosts including *Rhizobium* spp. (Stuurman et al., 2000) and *Agrobacterium* spp. For example, the pVS1 replicon is widely used in binary Ti vectors (Murai, 2013). Compared with genomic integration of genetic constructs, plasmids provide an advantage in terms of speed and convenience. Plasmids are also useful for complementation in *trans* and for genetic reporter systems (Andersen et al., 2001; Cheng et al., 2013), and provide an excellent platform for rapid screening of various genetic elements and reporter genes. These elements can later be incorporated into the genome, as has been shown in the present study using the mini-Tn7 transposon system. Additionally, expression of genetic elements can be regulated through control of plasmid copy number (Itoh et al., 1988), unlike genomic elements typically only present as a single copy. The necessity for maintaining selective pressure is a drawback to the use of plasmids, especially for *in vivo* studies where antibiotics may be detrimental to the model system. Some alternatives to antibiotics include auxotrophic markers, although these require strain engineering, or use of toxin/antitoxin systems. However, large, low copy number plasmids such as pVS1 typically contain replication and partition elements that ensure segregation to daughter cells (Thomas and Haines, 2004; Pinto et al., 2012; Funnell, 2016). In pME6031 these functions are provided by the *repA* and *parA* genes (**Figure 1**). A resolvase is also present, but is reportedly not required for stable maintenance (Heeb et al., 2000). Herein, we have built on the attributes of pME6031, adding constitutive promoters, next generation fluorescent proteins that function as bright, stable tags, and signal peptides that accommodate targeting of functional fluorescent proteins to the periplasmic space. Additionally, the combination of *P*_c_ or *P*_psbA_ promoters with a *repA* copy number mutation permits amplification of fluorescent protein expression levels over a modest range. We anticipate that these new genetic tools will enable rapid, bright and stable plasmid-based tagging of a variety of rhizosphere-associated bacterial strains. One cautionary note: the plasmids described herein retain an origin of transfer (*oriT*) that is part of the p15A replicon. While *oriT* is useful for enabling plasmid uptake via conjugation in difficult to transform strains, it should be inactivated before use in mixed populations or complex microbial communities where undesired plasmid mobilization could occur.

### Fluorescent Reporters

Many new fluorescent proteins have been described in recent years. These include improved variants of GFP and RFP derived from *Aequorea victoria* and *Discosoma* sp. as well as isolates from new species. Here we have selected a subset of favorable new variants that span the spectral range from cyan to far-red. Attributes considered for selection of fluorescent proteins included quantum yield, photostability, conformational stability, maturation rates and low self-association or aggregation tendency (Shaner et al., 2005). For studies of microbes in the rhizosphere, pKa of secreted fluorescent proteins is another important consideration, due to temporal and spatial dynamics of rhizosphere pH (Hinsinger et al., 2003; Blossfeld et al., 2013). Spectral characteristics of all fluorescent proteins used in this study are listed in **Table 4**. The cyan fluorescent protein, mTurquoise2 was selected for its high quantum yield, photostability and good maturation rates (Goedhart et al., 2012). It also boasts improved fluorescence decay kinetics that can enhance analysis of fluorescence lifetime measurements, and it is a superior FRET donor. mNeonGreen is extremely bright new fluorescent protein with a high quantum yield, fast maturation kinetics and stable monomeric characteristics (Shaner et al., 2013). It was evolved from a tetrameric yellow fluorescent protein from *Branchiostoma lanceolatum*, LanYFP, and has an excitation maximum intermediate between that of common GFPs and YFPs. mNeonGreen can serve as a FRET acceptor from mTurquoise2 and donor to red fluorescent proteins such as mRuby2. mRuby2 was selected as it is one of the brightest monomeric red fluorescent proteins available and has good photostability characteristics (Lam et al., 2012). Of note, a newer variant, mRuby3, has recently been described (Bajar et al., 2016), reportedly with improved brightness and photostability. This variant will be tested in future studies. Two tetrameric red and far-red fluorescent proteins were also incorporated into the vector suite. DsRed-Express2 and its derivative, E2-Crimson, were developed specifically as non-toxic red alternatives for labeling bacterial or mammalian cells (Strack et al., 2008, 2009). Because they are tetramers, these proteins are most applicable to whole cell labeling, and in the present study we have only expressed these proteins in the cytoplasm. When observed by confocal microscopy, DsRed-Express2 provided brighter labeling of *P. fluorescens* SBW25 cytoplasm than mRuby2.

### Constitutive Promoters for Fluorescent Protein Expression

For the present study, two different constitutive promoters, *P*_c_ and *P*_psbA_, were investigated. Promoter *P*_c_ is derived from the class 3 integron of *Delftia acidovorans* C17. Integrons are mobile genetic elements that are found in many Gram-negative, and some Gram-positive bacteria. Class 3 integrons have been found in pseudomonads and other genera; thus these promoters are predicted to work in a variety of microbial strains (Xu et al., 2013). *P*_c_ lies within the 5′ coding region of the integrase gene, and induces transcription of downstream elements (Xu et al., 2007). *P*_c_ has been reported to direct constitutive expression of reporter genes in several *Pseudomonas* strains, with levels twofold higher than *P_tac_* and about 3- and 50-fold higher, respectively, than constitutive *P_Tet_* and *P_Gm_* promoters (Xu et al., 2013). The second promoter tested was the chloroplast promoter *P*_psbA_, from *Amaranthus hybridus*, shown previously to function as a strong promoter in *P. fluorescens, P. syringae* and *A. tumefaciens* (Tombolini et al., 1997; Wang et al., 2007). Both *P*_c_ and *P*_psbA_ directed constitutive expression of all fluorescent proteins tested in *P. fluorescens* SBW25. Among the different *Pseudomonas* strains tested, the level of expression of mNeonGreen was somewhat higher (less than twofold difference) with *P*_psbA_ compared to *P*_c_ in liquid culture experiments. Interestingly, we observed that the *P*_psbA_ promoter region contains the translation start site for expression of the protein encoded by *psbA*, the D1 protein of the photosystem II reaction center (Hayashi et al., 2003). The start site is downstream of the predicted -35 and -10 regions, and we cannot rule out the possibility that a short segment of D1 is produced. In some cases, creation of a mini-cistron can enhance expression of the downstream genes (Skerra et al., 1991). We constructed two variants of *P*_psbA_ containing deletions of this region and observed no appreciable effect on expression of mNeonGreen in *P. fluorescens* SBW25 (Supplementary Figure S2), so in our studies, we have continued to use the full-length promoter element. In addition to the modest expression control provided by promoter selection, fluorescent protein production was also modulated by adjusting the copy number of the plasmid with the RepA A246V mutation. This mutation resulted in a 5 to 6-fold increase in expression of mNeonGreen in *P. fluorescens* SBW25. This increase is higher than might be expected based on the approximately twofold increase in plasmid copy number that the mutation produced in a *P. protogens* strain (Heeb et al., 2000), suggesting that the effect of the RepA mutation may be strain specific. We have not yet examined the effect of the RepA mutation in other *Pseudomonas* strains. In the present study, we have avoided using inducible promoters, because these are typically not suitable for plant-microbe model systems such as those under study in our lab, unless one wishes to link expression with presence of a particular solute or signal in the rhizosphere. However, in addition to screening different promoter elements, other factors, such as manipulation of the RBS, can be adjusted to regulate protein expression levels (Salis et al., 2009). Such modifications will be assessed in future iterations of the plasmids.

### Targeting Fluorescent Proteins to the Periplasm

The transport of functional GFP-family proteins to the bacterial periplasm is largely dependent on selection of an appropriate secretion pathway (Dammeyer and Tinnefeld, 2012). Choosing an incompatible pathway can result in premature folding in the cytoplasm or misfolding and aggregation in the periplasm. Studies have shown that co-translational secretion through the SRP-dependent SEC pathway or transport of fully folded protein through the Tat pathway can be effective for GFP export, depending on the particular GFP variant under study (Thomas et al., 2001; Aronson et al., 2011; Dinh and Bernhardt, 2011; Matos et al., 2012). Although native signal sequences have been successfully used for secretion of recombinant proteins in Pseudomonas (Retallack et al., 2007; Jin et al., 2011), for the present study we have used heterologous signal peptides from *E. coli* TorA and TorT proteins which are known to target Tat and SRP-dependent SEC pathways, respectively. While signal peptides are quite conserved through all domains of life, all having positively charged N-termini, hydrophobic cores, and polar C-termini, those targeting the aforementioned secretion pathways have unique properties. Signal peptides that target the SRP-dependent SEC pathway have increased hydrophobicity, and those that target Tat pathways have an additional, conserved ‘twin-arginine’ motif (Natale et al., 2008). The TorT signal peptide has a high calculated hydrophobicity, required for recognition by SRP (Lee and Bernstein, 2001; Huber et al., 2005). The TorA signal peptide has been used widely in *E. coli* to target GFP secretion through the Tat pathway (Thomas et al., 2001; Barrett et al., 2003; Speck et al., 2011). We tested secretion of the monomeric fluorescent proteins, mNeonGreen and mTurquoise2 by TorA and TorT under control of the *P*_c_ promoter. Both proteins were exported to the periplasm, but with different optimal signal peptides. We also compared secretion of the same proteins in *E. coli* DH5α, and observed qualitatively similar signal peptide specificity. Although these results are suggestive of export through *P. fluorescens* SBW25 SRP-Sec and Tat pathways, confirmation of this will require additional genetic tests. Nonetheless, the plasmids permit empirical selection of the best signal peptide for protein secretion.

mNeonGreen was exported most efficiently with the TorT signal peptide. Using the TorA peptide, high expression was achieved, but most of the protein remained trapped in the cytoplasm (**Figure 3A**). Although it is possible that mNeonGreen export was incompatible with the targeted pathway, this result could be due to pathway saturation. For example, in *E. coli* overexpression of Tat components has been shown to restore secretion or increase flux through this pathway (Barrett et al., 2003; Matos et al., 2012). On the contrary, mTurquoise2 export was most efficient with the TorA signal peptide. Although periplasmic localization could be observed using the TorT peptide, fluorescence was difficult to capture on the microscope. Likewise, in *E. coli* expression of mTurquoise2 with the TorT peptide produced barely visible fluorescence (**Figure 3B**).

### Stability of the Plasmids in Liquid Culture and in a Tomato Root Model System

Studies of the pME6031 vector indicated that it was stably maintained in *P. protegens* CHA0 for at least 100 generations. (Heeb et al., 2000) We confirmed these results in our laboratory and also compared stability of pME6031 in other *Pseudomonas* strains *in vitro*. Another *P. protegens* strain, Pf-5, showed fairly stable maintenance of the plasmid, and *P. fluorescens* strains SBW25 and Pf0-1 lost the plasmid at a slightly higher rate. *P. fluorescens* WH6 lost the plasmid fairly rapidly. Incorporation of the RepA mutation, A246V, improved stability (tested only in *P. fluorescens* SBW25). Plasmid loss rates can be overestimated in liquid culture experiments due to outcompeting by plasmid-free bacteria. In fact, in the hydroponic root culture system, colonization of fluorescent bacteria could be observed for more than 2 weeks, and double-labeled strains that expressed both genome-integrated and plasmid-borne fluorescent proteins confirmed plasmid stability. The increased plasmid retention in the rhizosphere could be due to a slowing of bacterial metabolism, as the root exudate is the only carbon source for bacterial growth in this system. It is also feasible that other factors contribute to plasmid stability in the unique environment of the root.

### Plasmid Distribution

Empty promoter plasmids and those expressing mTurquoise2, mRuby2, DsRed-Express2 and E2-Crimson will be distributed through Addgene^3^. Plasmids expressing mNeonGreen (under license from Allele Biotechnology, San Diego, CA, United States) will be provided to licensed users upon request. Please send requests to RW.

## Author Contributions

RW and KK conceived the experiments. RW, AA, and MB constructed the expression vectors. RW, AA, MB, and JJ analyzed fluorescent protein expression. JJ performed plasmid stability experiments. SS and RW designed and executed the root seedling colonization experiments. DS-D performed microscopy analysis of samples. RW wrote the paper draft. All authors read and approved the final manuscript.

## Conflict of Interest Statement

The authors declare that the research was conducted in the absence of any commercial or financial relationships that could be construed as a potential conflict of interest.

## Footnotes

1 http://www.blueheronbio.com/

2 http://www.compgen.org/tools/PRED-TAT/

3 www.addgene.org
