# Supplementary material for: A New Suite of Plasmid Vectors for Fluorescence-Based Imaging of Root Colonizing Pseudomonads
Source: Front Plant Sci. 2018 Feb 1;8:2242. doi: 10.3389/fpls.2017.02242 (PMC5799272; doi:10.3389/fpls.2017.02242)
Supplement: Supplementary file 1 [file Supplementary_Data.DOC]

LOCUS Exported 8247 bp ds-DNA circular SYN 08-DEC-2017

DEFINITION Sequence of plasmid pME6031 determined by Next Generation

Sequencing.

The annotations are from accession number AF118811.

NGS data file: RW003_pME6031_BG8_CIRCLE_8299_l1.

ACCESSION .

VERSION .

KEYWORDS .

SOURCE synthetic DNA construct

ORGANISM synthetic DNA construct

REFERENCE 1 (bases 1 to 8247)

AUTHORS Rosemarie Wilton, Angela J. Ahrendt, Shalaka V. Shinde, Deirdre J.

Sholto-Douglas, Jessica L. Johnson, Melissa B. Brennan, Kenneth M.

Kemner

TITLE A new suite of plasmid vectors for fluorescence-based imaging of

root colonizing pseudomonads

JOURNAL Exported Dec 8, 2017 from SnapGene 4.0.3 http://www.snapgene.com

REFERENCE 2 (bases 1 to 8247)

AUTHORS .

TITLE Direct Submission

JOURNAL Exported Dec 8, 2017 from SnapGene 4.0.3

http://www.snapgene.com

FEATURES Location/Qualifiers

source 1..8247

/organism="synthetic DNA construct"

/mol_type="other DNA"

CDS 194..880

/codon_start=1

/product="pVS1 resolvase"

/label=pVS1 resolvase

/note="ORF1; not required for stable maintenance"

/db_xref="GI:4378787"

/protein_id="AAD19683.1"

/translation="MNKSAAAGLLGYARVSTDDQDLTNQRAELHAAGCTKLFSEKITGT

RRDRPELARMLDHLRPGDVVTVTRLDRLARSTRDLLDIAERIQEAGAGLRSLAEPWADT

TTPAGRMVLTVFAGIAEFERSLIIDRTRSGREAAKARGVKFGPRPTLTPAQIAHARELI

DQEGRTVKEAAALLGVHRSTLYRALERSEEVTPTEARRRGAFREDALTEADALAAAENE

RQEEQA"

CDS 877..1092

/codon_start=1

/product="hypothetical protein"

/label=hypothetical protein

/note="ORF2; not required for stable maintenance"

/db_xref="GI:4378788"

/protein_id="AAD19684.1"

/translation="MKPHQDGQDEPFFITEEIEAEMIAAGYVFEPPAHVSTVRLHEILA

GLSDAKLAAWPASLAAEETERRRLKR"

CDS 1179..1808

/codon_start=1

/product="pVS1 partitioning protein"

/label=pVS1 partitioning protein

/note="StaA; similar to IncC2"

/db_xref="GI:4378789"

/protein_id="AAD19685.1"

/translation="MKVIAVLNQKGGSGKTTIATHLARALQLAGADVLLVDSDPQGSAR

DWAAVREDQPLTVVGIDRPTIDRDVKAIGRRDFVVIDGAPQAADLAVSAIKAADFVLIP

VQPSPYDIWATADLVELVKQRIEVTDGRLQAAFVVSRAIKGTRIGGEVAEALAGYELPI

LESRITQRVSYPGTAAAGTTVLESEPEGDAAREVQALAAEIKSKLI"

CDS 1829..2044

/codon_start=1

/product="hypothetical protein"

/label=hypothetical protein

/note="ORF3; not required for stable maintenance"

/db_xref="GI:4378790"

/protein_id="AAD19686.1"

/translation="MSKSTNTLSAGRPSARSSKAATLASLADTPAMKRVNFQLPAEDHT

KLKMYAVRQGKTITELLSEYIAQLPE"

CDS 2237..3310

/codon_start=1

/product="pVS1 replication protein"

/label=pVS1 replication protein

/note="RepA"

/db_xref="GI:4378791"

/protein_id="AAD19687.1"

/translation="MSGRKPSGPVQIGAALGDDLVEKLKAAQAAQRQRIEAEARPGESW

QAAADRIRKESRQPPAAGAPSIRKPPKGDEQPDFFVPMLYDVGTRDSRSIMDVAVFRLS

KRDRRAGEVIRYELPDGHVEVSAGPAGMASVWDYDLVLMAVSHLTESMNRYREGKGDKP

GRVFRPHVADVLKFCRRADGGKQKDDLVETCIRLNTTHVAMQRTKKAKNGRLVTVSEGE

ALISRYKIVKSETGRPEYIEIELADWMYREITEGKNPDVLTVHPDYFLIDPGIGRFLYR

LARRAAGKAEARWLFKTIYERSGSAGEFKKFCFTVRKLIGSNDLPEYDLKEEAGQAGPI

LVMRYRNLIEGEASAGS"

rep_origin 3376..3570

/label=pVS1 origin of replication

/note="pVS1 origin of replication"

rep_origin 4262..5091

/label=p15A origin of replication

/note="p15A origin of replication"

misc_feature 5066..5087

/label=p15A origin of transfer

/note="p15A origin of transfer"

terminator 5145..5411

/label=T4 transcription terminator

/note="T4 transcription terminator"

misc_feature 5413..5523

/label=multiple cloning site

/note="multiple cloning site"

CDS complement(6250..6927)

/codon_start=1

/product="RK2 tetracycline repressor protein"

/label=RK2 tetracycline repressor protein

/note="TetR"

/db_xref="GI:4378793"

/protein_id="AAD19689.1"

/translation="MFISDKVSSMTKLQPNTVIRAALDLLNEVGVDGLTTRKLAERLGV

QQPALYWHFRNKRALLDALAEAMLAENHTHSVPRADDDWRSFLIGNARSFRQALLAYRD

GARIHAGTRPGAPQMETADAQLRFLCEAGFSAGDAVNALMTISYFTVGAVLEEQAGDSD

AGERGGTVEQAPLSPLLRAAIDAFDEAGPDAAFEQGLAVIVDGLAKRRLVVRNVEGPRK

GDD"

CDS 6931..8205

/codon_start=1

/product="RK2 tetracycline resistance protein"

/label=RK2 tetracycline resistance protein

/note="TetA"

/db_xref="GI:4378792"

/protein_id="AAD19688.1"

/translation="MSTNLSVIKNPRVQSDQRRLVRRPDVKPNIPLIVILSTVALDAVG

IGLIMPVLPGLLRDLVHSNDVTAHYGILLALYALVQFACAPVLGALSDRFGRRPILLVS

LAGATVDYAIMATAPFLWVLYIGRIVAGITGATGAVAGAYIADITDGDERARHFGFMSA

CFGFGMVAGPVLGGLMGGFSPHAPFFAAAALNGLNFLTGCFLLPESHKGERRPLRREAL

NPLASFRWARGMTVVAALMAVFFIMQLVGQVPAALWVIFGEDRFHWDATTIGISLAAFG

ILHSLAQAMITGPVAARLGERRALMLGMIADGTGYILLAFATRGWMAFPIMVLLASGGI

GMPALQAMLSRQVDEERQGQLQGSLAALTSLTSIVGPLLFTAIYAASITTWNGWAWIAG

AALYLLCLPALRRGLWSGAGQRADR"

ORIGIN

1 gatccaaccc ctccgctgct atagtgcagt cggcttctga cgttcagtgc agccgtcttc

61 tgaaaacgac atgtcgcaca agtcctaagt tacgcgacag gctgccgccc tgcccttttc

121 ctggcgtttt cttgtcgcgt gttttagtcg cataaagtag aatacttgcg actagaaccg

181 gagacattac gccatgaaca agagcgccgc cgctggcctg ctgggctatg cccgcgtcag

241 caccgacgac caggacttga ccaaccaacg ggccgaactg cacgcggccg gctgcaccaa

301 gctgttttcc gagaagatca ccggcaccag gcgcgaccgc ccggagctgg ccaggatgct

361 tgaccaccta cgccctggcg acgttgtgac agtgaccagg ctagaccgcc tggcccgcag

421 cacccgcgac ctactggaca ttgccgagcg catccaggag gccggcgcgg gcctgcgtag

481 cctggcagag ccgtgggccg acaccaccac gccggccggc cgcatggtgt tgaccgtgtt

541 cgccggcatt gccgagttcg agcgttccct aatcatcgac cgcacccgga gcgggcgcga

601 ggccgccaag gcccgaggcg tgaagtttgg cccccgccct accctcaccc cggcacagat

661 cgcgcacgcc cgcgagctga tcgaccagga aggccgcacc gtgaaagagg cggctgcact

721 gcttggcgtg catcgctcga ccctgtaccg cgcacttgag cgcagcgagg aagtgacgcc

781 caccgaggcc aggcggcgcg gtgccttccg tgaggacgca ttgaccgagg ccgacgccct

841 ggcggccgcc gagaatgaac gccaagagga acaagcatga aaccgcacca ggacggccag

901 gacgaaccgt ttttcattac cgaagagatc gaggcggaga tgatcgcggc cgggtacgtg

961 ttcgagccgc ccgcgcacgt ctcaaccgtg cggctgcatg aaatcctggc cggtttgtct

1021 gatgccaagc tggcggcctg gccggccagc ttggccgctg aagaaaccga gcgccgccgt

1081 ctaaaaaggt gatgtgtatt tgagtaaaac agcttgcgtc atgcggtcgc tgcgtatatg

1141 atgcgatgag taaataaaca aatacgcaag gggaacgcat gaaggttatc gctgtactta

1201 accagaaagg cgggtcaggc aagacgacca tcgcaaccca tctagcccgc gccctgcaac

1261 tcgccggggc cgatgttctg ttagtcgatt ccgatcccca gggcagtgcc cgcgattggg

1321 cggccgtgcg ggaagatcaa ccgctaaccg ttgtcggcat cgaccgcccg acgattgacc

1381 gcgacgtgaa ggccatcggc cggcgcgact tcgtagtgat cgacggagcg ccccaggcgg

1441 cggacttggc tgtgtccgcg atcaaggcag ccgacttcgt gctgattccg gtgcagccaa

1501 gcccttacga catatgggcc accgccgacc tggtggagct ggttaagcag cgcattgagg

1561 tcacggatgg aaggctacaa gcggcctttg tcgtgtcgcg ggcgatcaaa ggcacgcgca

1621 tcggcggtga ggttgccgag gcgctggccg ggtacgagct gcccattctt gagtcccgta

1681 tcacgcagcg cgtgagctac ccaggcactg ccgccgccgg cacaaccgtt cttgaatcag

1741 aacccgaggg cgacgctgcc cgcgaggtcc aggcgctggc cgctgaaatt aaatcaaaac

1801 tcatttgagt taatgaggta aagagaaaat gagcaaaagc acaaacacgc taagtgccgg

1861 ccgtccgagc gcacgcagca gcaaggctgc aacgttggcc agcctggcag acacgccagc

1921 catgaagcgg gtcaactttc agttgccggc ggaggatcac accaagctga agatgtacgc

1981 ggtacgccaa ggcaagacca ttaccgagct gctatctgaa tacatcgcgc agctaccaga

2041 gtaaatgagc aaatgaataa atgagtagat gaattttagc ggctaaagga ggcggcatgg

2101 aaaatcaaga acaaccaggc accgacgccg tggaatgccc catgtgtgga ggaacgggcg

2161 gttggccagg cgtaagcggc tgggttgtct gccggccctg caatggcact ggaaccccca

2221 agcccgagga atcggcgtga gcggtcgcaa accatccggc ccggtacaaa tcggcgcggc

2281 gctgggtgat gacctggtgg agaagttgaa ggccgcgcag gccgcccagc ggcaacgcat

2341 cgaggcagaa gcacgccccg gtgaatcgtg gcaagcggcc gctgatcgaa tccgcaaaga

2401 atcccggcaa ccgccggcag ccggtgcgcc gtcgattagg aagccgccca agggcgacga

2461 gcaaccagat tttttcgttc cgatgctcta tgacgtgggc acccgcgata gtcgcagcat

2521 catggacgtg gccgttttcc gtctgtcgaa gcgtgaccga cgagctggcg aggtgatccg

2581 ctacgagctt ccagacgggc acgtagaggt ttccgcaggg ccggccggca tggccagtgt

2641 gtgggattac gacctggtac tgatggcggt ttcccatcta accgaatcca tgaaccgata

2701 ccgggaaggg aagggagaca agcccggccg cgtgttccgt ccacacgttg cggacgtact

2761 caagttctgc cggcgagccg atggcggaaa gcagaaagac gacctggtag aaacctgcat

2821 tcggttaaac accacgcacg ttgccatgca gcgtacgaag aaggccaaga acggccgcct

2881 ggtgacggta tccgagggtg aagccttgat tagccgctac aagatcgtaa agagcgaaac

2941 cgggcggccg gagtacatcg agatcgagct agctgattgg atgtaccgcg agatcacaga

3001 aggcaagaac ccggacgtgc tgacggttca ccccgattac tttttgatcg atcccggcat

3061 cggccgtttt ctctaccgcc tggcacgccg cgccgcaggc aaggcagaag ccagatggtt

3121 gttcaagacg atctacgaac gcagtggcag cgccggagag ttcaagaagt tctgtttcac

3181 cgtgcgcaag ctgatcgggt caaatgacct gccggagtac gatttgaagg aggaggcggg

3241 gcaggctggc ccgatcctag tcatgcgcta ccgcaacctg atcgagggcg aagcatccgc

3301 cggttcctaa tgtacggagc agatgctagg gcaaattgcc ctagcagggg aaaaaggtcg

3361 aaaaggtctc tttcctgtgg atagcacgta cattgggaac ccaaagccgt acattgggaa

3421 ccggaacccg tacattggga acccaaagcc gtacattggg aaccggtcac acatgtaagt

3481 gactgatata aaagagaaaa aaggcgattt ttccgcctaa aactctttaa aacttattaa

3541 aactcttaaa acccgcctgg cctgtgcata actgtctggc cagcgcacag ccgaagagct

3601 gcaaaaagcg cctacccttc ggtcgctgcg ctccctacgc cccgccgctt cgcgtcggcc

3661 tatcgcggcc gctggccgct caaaaatggc tggcctacgg ccaggcaatc taccagggcg

3721 cggacaagcc gcgccgtcgc cactcgaccg ccggcgctga ggtctgcctc gtgaagaagg

3781 tgttgctgac tcataccagg caatggcaac aacgttgcgc aaactattaa ctggcgaact

3841 acttactcta gcttcccggc aacaattaat agactggatg gaggcggata aagttgcagg

3901 accacttctg cgctcggccc ttccggctgg ctggtttatt gctgataaat ctggagccgg

3961 tgagcgtggg tctcgcggta tcattgcagc actggggcca gatggtaagc cctcccgtat

4021 cgtagttatc tacacgacgg ggagtcaggc aactatggat gaacgaaata gacagatcgc

4081 tgagataggt gcctcactga ttaagcattg gtaactgtca gaccaagttt actcatatat

4141 actttagatt gatttaaaac ttcattttta atttaaaagg atctaggtga agatcctttt

4201 tgataatctc atgaccaaaa tcccttaacg tgagttttcg ttccactgag cgtcagaccc

4261 cttaataaga tgatcttctt gagatcgttt tggtctgcgc gtaatctctt gctctgaaaa

4321 cgaaaaaacc gccttgcagg gcggtttttc gaaggttctc tgagctacca actctttgaa

4381 ccgaggtaac tggcttggag gagcgcagtc accaaaactt gtcctttcag tttagcctta

4441 accggcgcat gacttcaaga ctaactcctc taaatcaatt accagtggct gctgccagtg

4501 gtgcttttgc atgtctttcc gggttggact caagacgata gttaccggat aaggcgcagc

4561 ggtcggactg aacggggggt tcgtgcatac agtccagctt ggagcgaact gcctacccgg

4621 aactgagtgt caggcgtgga atgagacaaa cgcggccata acagcggaat gacaccggta

4681 aaccgaaagg caggaacagg agagcgcacg agggagccgc cagggggaaa cgcctggtat

4741 ctttatagtc ctgtcgggtt tcgccaccac tgatttgagc gtcagatttc gtgatgcttg

4801 tcaggggggc ggagcctatg gaaaaacggc tttgccgcgg ccctctcact tccctgttaa

4861 gtatcttcct ggcatcttcc aggaaatctc cgccccgttc gtaagccatt tccgctcgcc

4921 gcagtcgaac gaccgagcgt agcgagtcag tgagcgagga agcggaatat atcctgtatc

4981 acatattctg ctgacgcacc ggtgcagcct tttttctcct gccacatgaa gcacttcact

5041 gacaccctca tcagtgccaa catagtaagc cagtatacac tccgctagcg ctgaggtctg

5101 cctcgtgaag aaggtgttgc tgactcatac caggtagtcc gaggagcttt atgcttgtaa

5161 accgttttgt gaaaaaattt ttaaaataaa aaaggggacc tctagggtcc ccaattaatt

5221 agtaatataa tctattaaag gtcattcaaa aggtcatcca ccggatcaat tcccctgctc

5281 gcgcaggctg ggtgccaagc tctcgggtaa catcaaggcc cgatccttgg agcccttgcc

5341 ctcccgcacg atgatcgtgc cgtgatcgaa atccagatcc ttgacccgca gttgcaaacc

5401 ctcactgatc cgctagtccg aggcctcgag atctatcgat gcatgccatg gtacccggga

5461 gctcgaattc gaagcttctg cagacgcgtc gacgtcatat ggatccgata tcgccgtggc

5521 ggccagcttt tgccattctc accggattca gtcgtcactc atggtgattt ctcacttgat

5581 aaccttattt ttgacgaggg gaaattaata ggttgtattg atgttggacg agtcggaatc

5641 gcagaccgat accaggatct tgccatccta tggaactgcc tcggtgagtt ttctccttca

5701 ttacagaaac ggctttttca aaaatatggt attgataatc ctgatatgaa taaattgcag

5761 tttcatttga tgctcgatga gtttttctaa tcagaattgg ttaattggtt gtaacactgg

5821 cagagcatta cgctgacttg acgggacggc ggctttgttg aataaatcga acttttgctg

5881 agttgaagga tcagatcacg catcttcccg acaacgcaga ccgttccgtg gcaaagcaaa

5941 agttcaaaat caccaactgg tccacctaca acaaagctct catcaaccgt ggctccctca

6001 ctttctggct ggatgatggg gcgattcagg cctggtatga gtcagcaaca ccttcttcac

6061 gaggcagacc tcagcgccag aaggccgcca gagaggccga gcgcggccgt gaggcttgga

6121 cgctagggca gggcatgaaa aagcccgtag cgggctgcta cgggcgtctg acgcggtgga

6181 aagggggagg ggatgttgtc tacatggctc tgctgtagtg agtgggttgc gctccggcag

6241 cggtcctgat caatcgtcac cctttctcgg tccttcaacg ttcctgacaa cgagcctcct

6301 tttcgccaat ccatcgacaa tcaccgcgag tccctgctcg aacgctgcgt ccggaccggc

6361 ttcgtcgaag gcgtctatcg cggcccgcaa cagcggcgag agcggagcct gttcaacggt

6421 gccgccgcgc tcgccggcat cgctgtcgcc ggcctgctcc tcaagcacgg ccccaacagt

6481 gaagtagctg attgtcatca gcgcattgac ggcgtccccg gccgaaaaac ccgcctcgca

6541 gaggaagcga agctgcgcgt cggccgtttc catctgcggt gcgcccggtc gcgtgccggc

6601 atggatgcgc gcgccatcgc ggtaggcgag cagcgcctgc ctgaagctgc gggcattccc

6661 gatcagaaat gagcgccagt cgtcgtcggc tctcggcacc gaatgcgtat gattctccgc

6721 cagcatggct tcggccagtg cgtcgagcag cgcccgcttg ttcctgaagt gccagtaaag

6781 cgccggctgc tgaaccccca accgttccgc cagtttgcgt gtcgtcagac cgtctacgcc

6841 gacctcgttc aacaggtcca gggcggcacg gatcactgta ttcggctgca actttgtcat

6901 gcttgacact ttatcactga taaacataat atgtccacca acttatcagt gataaagaat

6961 ccgcgcgttc aatcggacca gcggaggctg gtccggaggc cagacgtgaa acccaacata

7021 cccctgatcg taattctgag cactgtcgcg ctcgacgctg tcggcatcgg cctgattatg

7081 ccggtgctgc cgggcctcct gcgcgatctg gttcactcga acgacgtcac cgcccactat

7141 ggcattctgc tggcgctgta tgcgttggtg caatttgcct gcgcacctgt gctgggcgcg

7201 ctgtcggatc gtttcgggcg gcggccaatc ttgctcgtct cgctggccgg cgccactgtc

7261 gactacgcca tcatggcgac agcgcctttc ctttgggttc tctatatcgg gcggatcgtg

7321 gccggcatca ccggggcgac tggggcggta gccggcgctt atattgccga tatcactgat

7381 ggcgatgagc gcgcgcggca cttcggcttc atgagcgcct gtttcgggtt cgggatggtc

7441 gcgggacctg tgctcggtgg gctgatgggc ggtttctccc cccacgctcc gttcttcgcc

7501 gcggcagcct tgaacggcct caatttcctg acgggctgtt tccttttgcc ggagtcgcac

7561 aaaggcgaac gccggccgtt acgccgggag gctctcaacc cgctcgcttc gttccggtgg

7621 gcccggggca tgaccgtcgt cgccgccctg atggcggtct tcttcatcat gcaacttgtc

7681 ggacaggtgc cggccgcgct ttgggtcatt ttcggcgagg atcgctttca ctgggacgcg

7741 accacgatcg gcatttcgct tgccgcattt ggcattctgc attcactcgc ccaggcaatg

7801 atcaccggcc ctgtagccgc ccggctcggc gaaaggcggg cactcatgct cggaatgatt

7861 gccgacggca caggctacat cctgcttgcc ttcgcgacac ggggatggat ggcgttcccg

7921 atcatggtcc tgcttgcttc gggtggcatc ggaatgccgg cgctgcaagc aatgttgtcc

7981 aggcaggtgg atgaggaacg tcaggggcag ctgcaaggct cactggcggc gctcaccagc

8041 ctgacctcga tcgtcggacc cctcctcttc acggcgatct atgcggcttc tataacaacg

8101 tggaacgggt gggcatggat tgcaggcgct gccctctact tgctctgcct gccggcgctg

8161 cgtcgcgggc tttggagcgg cgcagggcaa cgagccgatc gctgatcgtg gaaacgatag

8221 gggccgctct agaactagct agaacta

//
