# Supplementary material for: A New Suite of Plasmid Vectors for Fluorescence-Based Imaging of Root Colonizing Pseudomonads
Source: Front Plant Sci. 2018 Feb 1;8:2242. doi: 10.3389/fpls.2017.02242 (PMC5799272; doi:10.3389/fpls.2017.02242)
Supplement: Supplementary file 2 [file Supplementary_Figures_and_Tables.PDF]

Supplementary Material

A new suite of plasmid vectors for fluorescence-based imaging of root colonizing pseudomonads

Rosemarie Wilton*, Angela J. Ahrendt, Shalaka Shinde, Deirdre J. Sholto-Douglas, Jessica L. Johnson, Melissa B. Brennan, Kenneth M. Kemner

*** Correspondence:** rwilton@anl.gov

Supplementary Tables

Table S1. Mutagenic primers used to remove restriction sites and incorporate the RepA A246V mutation. Mutation sites are underlined.

**Primer Name**	**Sequence**
pME6031_NotI_844_F	5'-CGACGCCCTGGCAGCCGCCGAGAATG-3'
pME6031_NotI_2377_F	5'-GTGAATCGTGGCAAGCAGCCGCTGATCGAATCC-3'
pME6031_NotI_3667_F	5'-CCTATCGCGGCCGATGGCCGCTCAAAAATGG-3'
pME6031_Bam/XbaI_F	5'-GGCCGCTCTAGTACTAGCTAGAACTATGATCCAACCCCTC-3'
RepA_A246V.For	5'-CATCGAGATCGAGCTAGTTGATTGGATGTACCGC-3'
RepA_A246V.Rev	5'-GCGGTACATCCAATCAACTAGCTCGATCTCGATG-3'

Table S2. Sequencing primers for promoters, fluorescent protein inserts and vector mutations.

Primer Name	SEQUENCE	Comments
PcPRO	5'-GGATGTCGAAACCCACCAAG-3'	Forward primer that anneals within the P_c promoter region to allow sequence analysis of downstream fluorescent protein inserts
PsbPRO	5'-TGACACGGGCGTATAAGACA-3'	Forward primer that anneals within the *psbA* promoter region to allow sequence analysis of downstream fluorescent protein inserts
T4Term	5'-AGATCCTTGACCCGCAGTTG-3'	Reverse primer that anneals in the T4 terminator region
MCSFor	5'-CTGAATCCGGTGAGAATGGC-3'	Forward primer that anneals upstream of the MCS to capture the full promoter region
NotI_3677.For	5'-CTATTAATTGTTGCCGGGAA CTAGAG-3'	For verifying mutation
NotI_2377.For	5'-AAAACGGCCACGTCCATGAT-3'	For verifying mutation
NotI_844.For	5'-AAATACACATCACCTTTTTAGACGGCG-3'	For verifying mutation
XbaI_8291/BamHI_1.For	5'-ACTTTATGCGACTAAAACACGCGAC-3'	For verifying mutation
RepA_Seq.For	5'-TACGACCTGGTACTGATGGC-3'	For verifying mutation

Table S3. Sequences of synthetic DNA constructs coding for promoters, signal peptides and codon-optimized fluorescent proteins. Restriction sites are underlined. Promoter -10 and -35 regions and the RBS are highlighted in grey and predicted transcription start sites are shown in bold. Signal peptide sequences are given in lower case. A potential start site for translation of a PsbA protein fragment is indicated by a black box and the cryptic RBS is italicized (Kim and Mullet, 1994). Identification of -35, -10 and transcription start sites for the P_c and *psbA* promoters is from Collis et al. (Collis et al., 2002) and Hayashi et al.(Hayashi et al., 2003), respectively.

>gBlock Pc_TorT_mTQ2

CCATGAATACGCGTTGCCGATACAAGAACAACAGCGCGTTGAGCGCCTGCCGGTGGGTGGCCGGCGCCACTTGCTTC
TCGGTGGCGAGCATGGTCAGAAAACCCTCGACTTCAGCTTGCCCCATTTCGCGCGGATGTCGAAACCCACCAAGGCT
GCGGGCCGTCCACAACACAAATGCCTTGGCCCAGTAGACATAAGCCTTCTCGGTCTGTAGGCTGTAAT**GC**AGGTAGC
GAACCCGTTCAAGAAATAATTTTGTTTAACTTTAAGAAGGAGATATACATatgcgcgtactgctatttttacttctttccctttt catgttgccggcatt
ttcgGCGAATTCCAAGGGCGAAGAGCTCTTTACCGGCGTTGTGCCAATCCTCGTGGAACTCGATGGCGACGTGAATGGC
CATAAATTTTCCGTGAGCGGCGAAGGCGAGGGCGATGCAACTTACGGCAAACTGACCCTGAAATTTATCTGCACTAC
CGGCAAACTCCCTGTTCCTTGGCCTACCCTCGTGACCACCTTGAGCTGGGGCGTCCAATGCTTTGCCCGTTACCCCGA
CCATATGAAACAACACGATTTCTTCAAAAGCGCCATGCCAGAAGGCTATGTTCAAGAACGCACCATTTTCTTCAAAG
ACGACGGCAACTACAAAACTCGGGCCGAAGTGAAATTCGAAGGCGATACCCTGGTCAACCGCATCGAACTGAAGGG
CATTGATTTTAAGGAAGACGGTAACATCCTGGGCCACAAACTCGAATACAACTATTTCTCCGACAACGTTTACATCAC
CGCTGACAAACAGAAAAACGGCATCAAAGCTAATTTTAAAATCCGCCATAATATCGAAGACGGCGGTGTTCAACTCG
CAGACCATTATCAACAAAATACCCCCATCGGCGACGGCCCCGTTCTGCTGCCTGACAACCATTACCTCTCCACCCAAA
GTAAACTCAGTAAAGATCCTAACGAAAAACGCGACCACATGGTTCTGCTGGAGTTCGTGACCGCGGCCGGCATCACC
CTGGGCATGGACGAGCTGTACAAGGCGTAAGCGGCCGCACATCATCATCACCATCACTAAGCTTTAAGCATCTCGAG
TTACACCGATACTATGG

>gBlock Pc_TorA_mRuby2

CCATGAATACGCGTTGCCGATACAAGAACAACAGCGCGTTGAGCGCCTGCCGGTGGGTGGCCGGCGCCACTTGCTTC
TCGGTGGCGAGCATGGTCAGAAAACCCTCGACTTCAGCTTGCCCCATTTCGCGCGGATGTCGAAACCCACCAAGGCT
GCGGGCCGTCCACAACACAAATGCCTTGGCCCAGTAGACATAAGCCTTCTCGGTCTGTAGGCTGTAAT**GC**AGGTAGC
GAACCCGTTCAAGAAATAATTTTGTTTAACTTTAAGAAGGAGATATACATatgaacaataacgatctctttcaggcatcacgtcggcgttttctgg
cacaactcggcggcttaaccgtcgccgggatgctggggccgtcattgttaacgccgcgacgtgcgactgcggcgcaagcgGCGAATTCCAAAGGTGAAGAACTGAT
CAAAGAAAACATGCGCATGAAAGTGGTGATGGAAGGTAGCGTGAACGGTCACCAATTCAAATGCACCGGCGAGGGC
GAAGGCAACCCCTACATGGGCACCCAAACCATGCGGATTAAGGTGATTGAAGGCGGGCCCCTCCCTTTCGCATTCGA
TATTCTCGCGACCTCCTTTATGTACGGCTCGCGCACGTTTATCAAGTACCCCAAGGGCATCCCGGATTTCTTCAAGCA
GTCCTTTCCAGAAGGTTTCACGTGGGAACGGGTTACCCGTTACGAGGACGGTGGCGTCGTTACTGTCATGCAAGACA
CTTCCCTCGAAGATGGTTGCCTGGTGTATCATGTGCAAGTCCGCGGTGTTAACTTTCCGAGTAACGGCCCTGTGATGC
AAAAGAAGACCAAAGGTTGGGAACCGAATACCGAAATGATGTATCCCGCTGACGGTGGCCTGCGCGGCTATACTCA
CATGGCGTTGAAAGTGGACGGCGGCGGCCATTTGTCGTGCAGTTTCGTCACTACTTATCGTTCCAAAAAGACGGTTGG
CAATATCAAAATGCCCGGGATTCACGCAGTCGATCATCGTTTGGAGCGCCTGGAAGAAAGCGACAACGAGATGTTCG
TCGTGCAACGTGAACACGCTGTGGCCAAATTTGCGGGCTTGGGCGGTGGTATGGATGAGTTGTATAAATAAGCGGCC
GCACATCATCATCACCATCACTAAGCTTTAAGCATCTCGAGTTACACCGATACTATGG

>gBlock PpsbA

CCATGAATACGCGTGGATCTCAATGAATATTGGTTGACACGGGCGTATAAGACATGTTATACTGTTG**A**ATAACAAGC
TATCAATTCTCTATTTTGAGAAGAATTAATGTGCTTG*GGAG*TCCCTGATGATTAAATTAAATAAACCAAGATTTTACC
ATGACTGCAATTTTAGAGAGACGGGGATCGATCCTCTTGAAATAATTTTGTTTAACTTTAAGAAGGAGATATACATA
TGAATTCCAAA

>gBlock_mNeonGreen

ATGAATTCGAAAGGCGAAGAAGATAACATGGCCAGTTTACCGGCAACTCATGAATTACATATTTTTGGAAGTATTAA
CGGCGTTGACTTTGATATGGTTGGACAGGGCACCGGCAACCCGAACGATGGTTATGAAGAATTAAACCTGAAATCTA
CAAAAGGCGACCTGCAATTCTCTCCCTGGATTCTTGTTCCACACATTGGTTATGGCTTCCACCAATACTTACCCTATCC
TGATGGCATGTCCCCTTTCCAAGCCGCGATGGTGGATGGCTCTGGTTACCAAGTACACCGTACCATGCAATTCGAAGA
TGGAGCATCACTCACAGTAAACTACCGCTATACATATGAAGGTTCCCACATTAAAGGCGAAGCACAAGTTAAAGGTA
CTGGATTCCCCGCTGATGGACCAGTTATGACTAACTCATTGACCGCAGCAGACTGGTGTCGCTCAAAAAAAACCTAT
CCGAACGATAAAACGATTATTAGCACATTTAAATGGAGTTATACGACCGGAAATGGCAAACGTTACCGCAGCACAGC
ACGCACAACCTATACATTTGCAAAACCAATGGCCGCGAATTATTTGAAAAACCAACCGATGTATGTCTTTCGTAAAA
CAGAATTAAAACACAGCAAAACAGAATTAAATTTTAAAGAATGGCAAAAAGCATTTACCGACGTTATGGGAATGGA
TGAATTATATAAATAAGCGGCCGCACATCATCATCACCATCACTAAGCTTTAAGCATCTCGAGTTACACCGATACTAT
GG

Table S4. Oligonucleotides used for PCR amplification and construction of fluorescent protein expression vectors. Restriction sites are underlined.

Primer name	Sequence	Comments
MluI-Pc.FOR	5’-CCATGAATACGCGTTGCCGATAC-3’	Forward amplification of P_c promoter; for cloning into MluI site of vector
MluI-PsbA.FOR	5’-CCATGAATACGCGTGGATCTCAATG-3’	Forward amplification of *psbA* promoter; for cloning into MluI site of vector
EcoRI-Pro.REV	5’-AGTCAGGAATTCATATGTATATCTCCTT CTTAAAGTTAAACAAAATTATTTC-3’	Reverse amplification of P_c or *psbA* promoter; for cloning into EcoRI site of vector
EcoRI-TorA.REV	5’-ATGCATGAATTCGCCGCTTGCGCC-3’	Reverse amplification of TorA signal peptide; for cloning into EcoRI site of vector
EcoRI-TorT.REV	5’-ATGCATGAATTCGCCGAAAATGCCGGC-3’	Reverse amplification of TorT signal peptide; for cloning into EcoRI site of vector
XhoI-FP.REV	5’-CCATAGTATCGGTGTAACTCGAGATGC-3’	Reverse amplification of constructs containing the His-tag segment; for cloning into XhoI site of vector.
EcoRI-mTQ2.FOR	5’-ATCTAGATGAATTCCAAGGGCGAAGAGCTCTTTACCG-3’	Forward amplification of mTurquoise2; for cloning into EcoRI site.
EcoRI-mNG.FOR	5’-ATCGATAGAATTCGAAAGGCGAAGAAGATAACATGG-3’	Forward amplification of mNeonGreen; for cloning into EcoRI site.
EcoRI-mRuby2.FOR	5’-AGTCACGAATTCCAAAGGTGAAGAACTGATCAAAGAAAAC-3’	Forward amplification of mRuby2; for cloning into EcoRI site.
EcoRI-DsRED.FOR	5’-ATCTAGATGAATTCCACTGAGAACGTCATCAAGCCC-3’	Forward amplification of DsRed-Express2 and E2-Crimson; for cloning into EcoRI site.
XhoI-DsRED.REV	5’-TCAGATCTCGAGTTACTACTGGAACAGGTGGTGGCG-3’	Reverse amplification of DsRed-Express2 and E2-Crimson; for cloning into XhoI site. Incorporates stop codon from source vector; no His-tag in resulting plasmids.
mTn7vecF1	5’-GAGCTCATGCATGATCGAATTAGCTTC-3’	Mini-Tn7 vector amplification and Seamless Cloning.
mTn7vecR1	5’-TCGCGAAGGCCTTGCAGG-3’	Mini-Tn7 vector amplification and Seamless Cloning.
PcInsertF	5’-GCAAGGCCTTCGCGAGCCGATACAAGAACAACAGCGC-3’	Forward primer for amplification of Pc-Fluorescent Protein-T4Term and Seamless Cloning
T4TermAmpR	5’-ATCATGCATGAGCTCTGTTGCTGACTCATACCAGGCTAG-3’	Reverse primer for amplification of Pc-Fluorescent Protein-T4Term and Seamless Cloning
GlmSFA	5’-CCGATCCTCTACACCATCCC-3’	SBW25-specific forward primer for amplifying the mini-Tn7 insertion site.
GlmSRA	5’-CAGTTCGATGGTATCCGCAC-3’	SBW25-specific reverse primer for amplifying the mini-Tn7 insertion site.

Table S5. Plant Nutrient Solution

The table indicates the final concentrations of each compound in the hydroponic plant growth medium (Desai et al., 2014). The pH was adjusted to 5.7 with 0.1 N HCl or 0.1 N KOH as needed, and the solution was sterilized by autoclaving.

**Compound**	**FC (µM)**
$Ca(NO_3)_2·4H_2O$	200.0
$CoCl_2·6H_2O$	0.5
$CuSO_4·5H_2O$	0.5
Fe,Na-EDTA	20.0
H_3BO_3	25.0
KCl	44.0
KH_2PO_4	50.0
KNO_3	400.0
$MgSO_4·7H_2O$	100.0
$MnSO_4·H_2O$	2.0
Na_2MoO_4	0.5
NH_4NO_3	402.3
$ZnSO_4·7H_2O$	2.0

Supplementary Figures

Figure S1. Hydroponic growth of tomato seedlings in conical centrifuge tubes. The tubes shown are 15 ml conical centrifuge tubes; 50 ml tubes can also be used to minimize demand for Nutrient Solution replenishment.

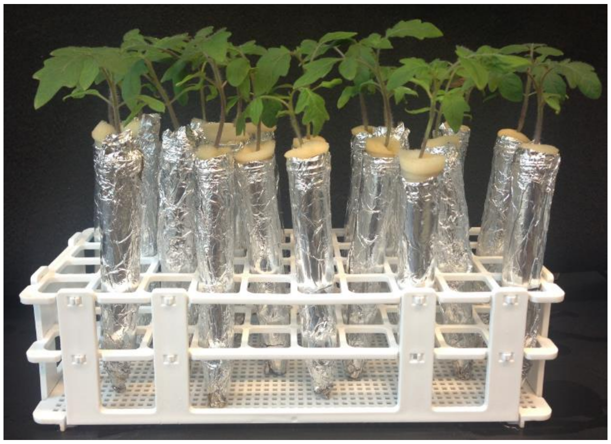

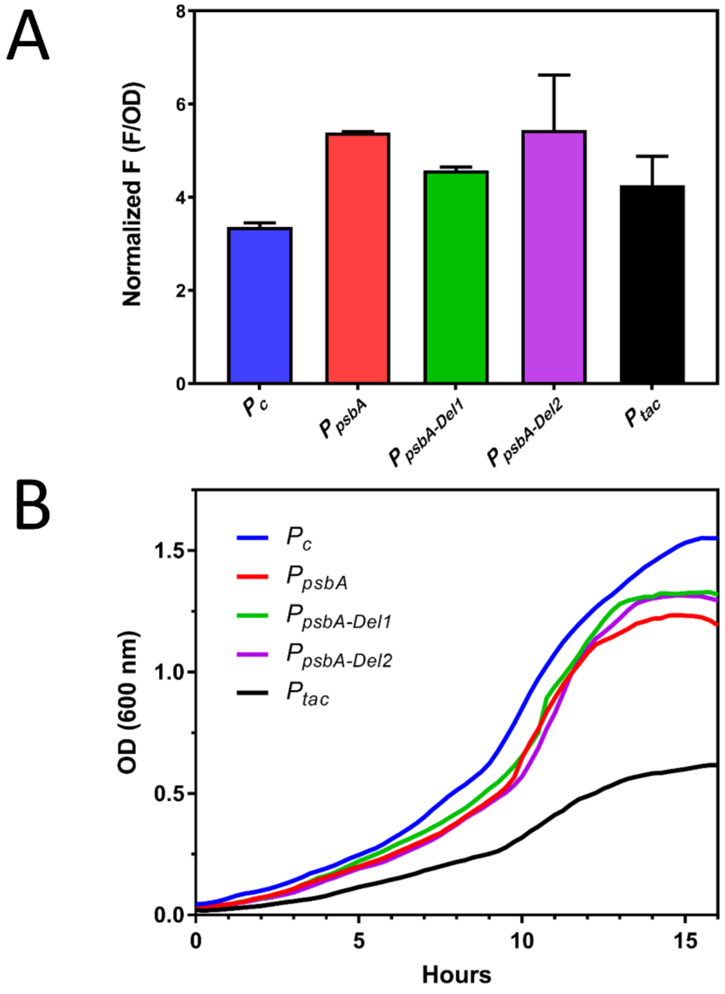

Figure S2. **(A)** Comparison of mNeonGreen expression levels from P_{psbA} deletion variants and P_{tac}. The plasmid in each case is the low-copy variant, pSW002 containing the indicated promoter upstream of mNeonGreen. Expression was tested in *P. fluorescens* SBW25. For reference, expression driven by P_c is also shown. Each histogram bar depicts the mean and standard deviation of the normalized fluorescence generated from two independent starter cultures. Three replicate wells were averaged for each culture. Fluorescence values at early stationary phase were normalized according to culture optical density at 600 nm. **(B)** Effect of promoter variants on culture growth kinetics of *P. fluorescens* SBW25. Representative growth curves are shown, selected from data sets used to generate the top figure.

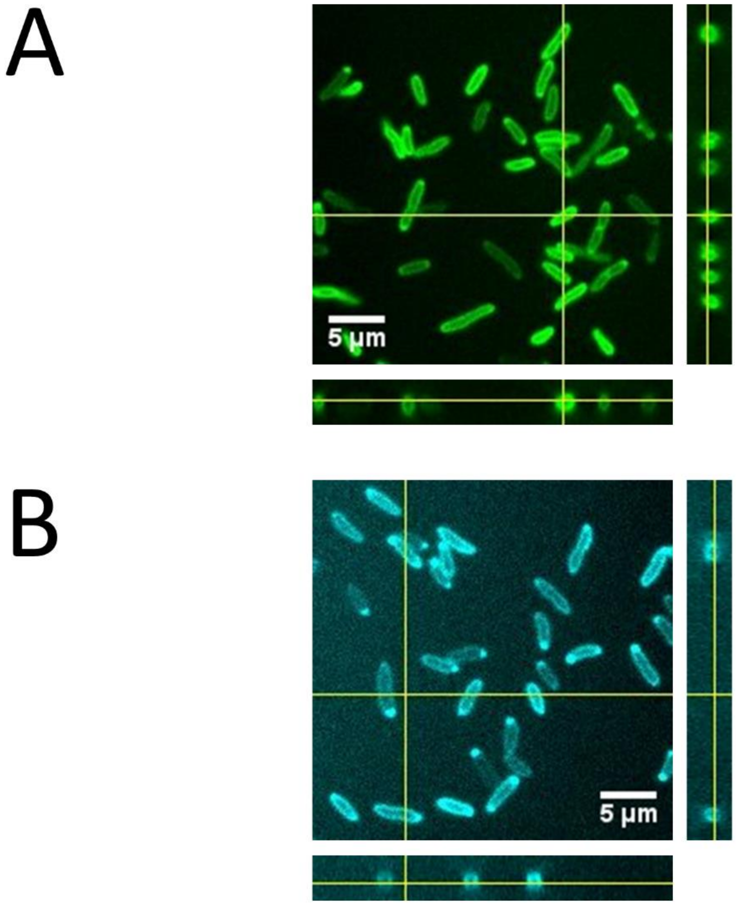

Figure S3. Confocal images with orthogonal views demonstrate the localization of fluorescent proteins in the periplasm of *P. fluorescens* SBW25. **(A)** mNeonGreen was secreted into the periplasmic space with the TorT signal peptide. **(B)** mTurquoise2 was secreted with the TorA signal peptide. In each image, the lower segment represents the X/Z plane, and the right segment represents the Y/Z plane. The yellow crosshairs indicate the location of each plane. Plasmid pSW002 with P_c promoter was used for expression.

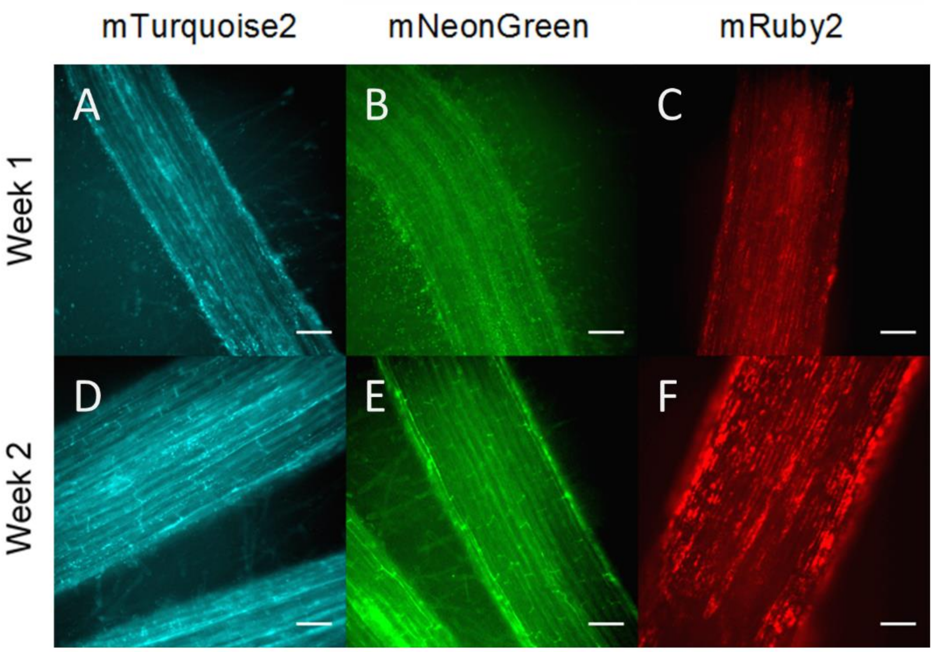

Figure S4. Low magnification (10x objective) confocal analysis of tomato seedling (*Solanum lycopersicum*) colonization by *P. fluorescens* SBW25 expressing monomeric fluorescent proteins. Bacteria are expressing mTurquoise2, mNeonGreen or mRuby2 in the cytoplasm (no signal peptide) and driven by the P_c promoter in plasmid pSW002. **(A-C)** Confocal images of root sections taken one week post inoculation. **(D-F)** Two weeks post inoculation. The scale bars are 100 µm long. Bacterial fluorescence is visible in crevices between root rhizodermal cells. Additional delineation of root structure is due to autofluorescence.

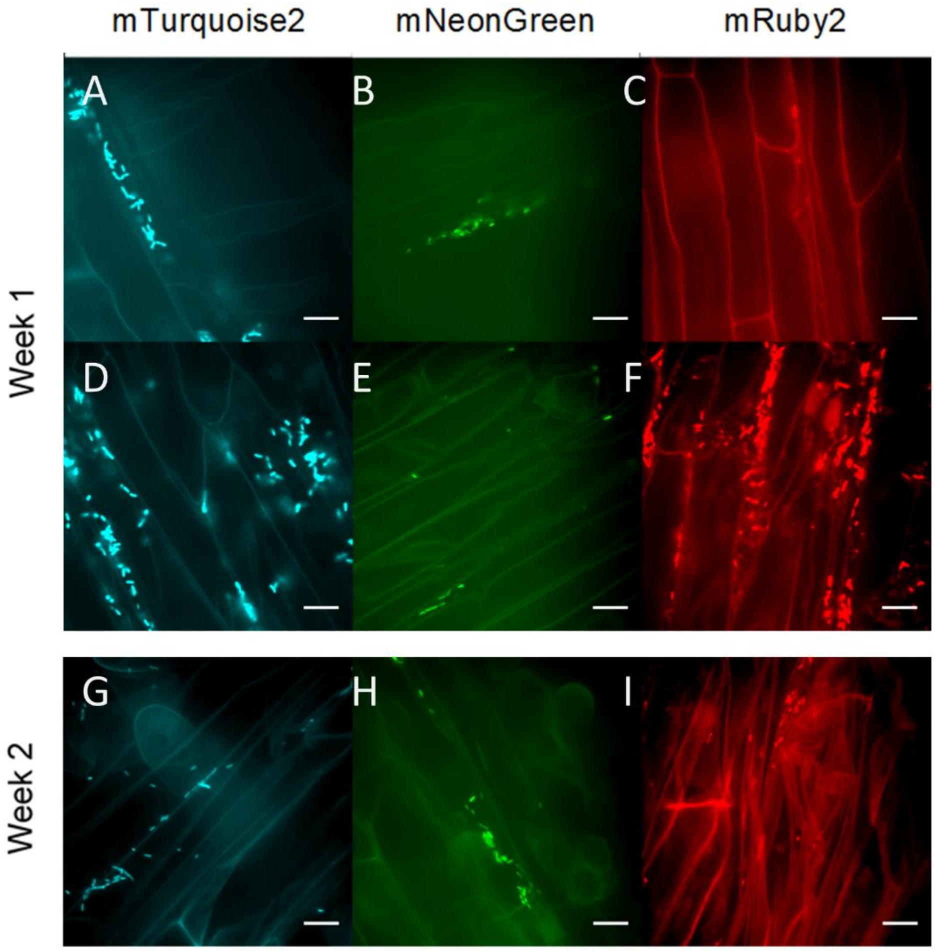

Figure S5. High magnification (100x objective) confocal analysis of tomato seedling (*Solanum lycopersicum*) colonization by *P. fluorescens* SBW25 expressing monomeric fluorescent proteins. Bacteria are expressing mTurquoise2, mNeonGreen or mRuby2 in the cytoplasm (no signal peptide), driven by the P_c promoter in plasmid pSW002. **(A-F)** Confocal images of root sections taken one week post inoculation. **(G-I)** Two weeks post inoculation; to augment visual analysis of bacterial colonization, figures g-i are displayed as maximum intensity projections of confocal Z stacks. Slice depth 0.2 µm; **(G)** 7 µm, **(H)** 5.4 µm, **(I)** 1.8 µm. The scale bars are 10 µm long. Delineation of root structure is due to autofluorescence.

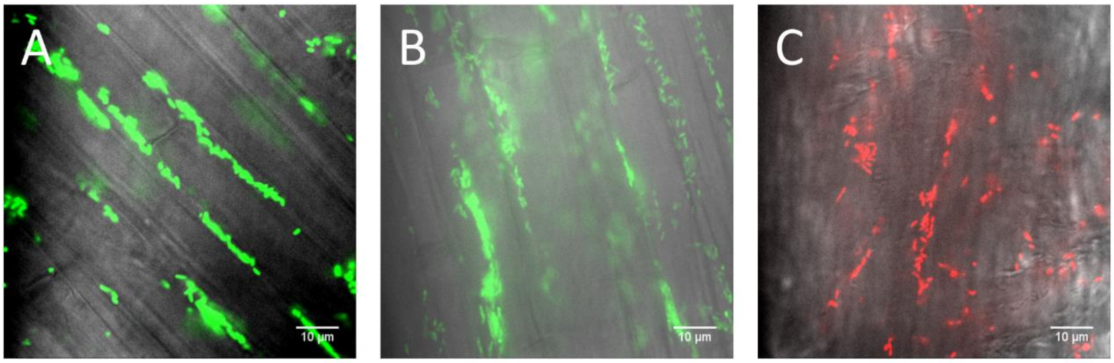

Figure S6. Overlay of confocal and brightfield images showing colonization of *P. fluorescens* SBW25 expressing mNeonGreen or DsRed-Express2. The images were obtained 16 days post inoculation. (A) High copy number plasmid pSW003-P_c-mNeonGreen, (B) plasmid pSW002-P_c-mNeonGreen, (C) plasmid pSW002-P_c-DsRed-Express2.

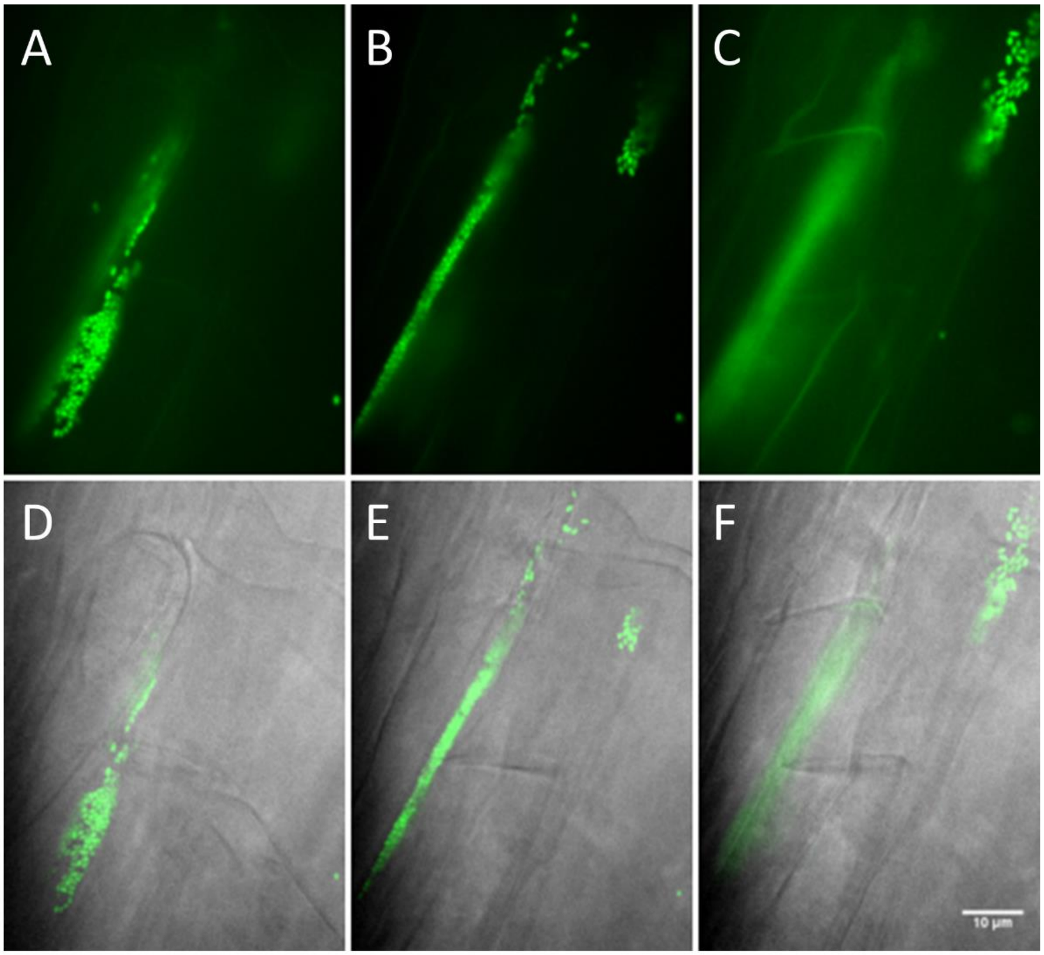

Figure S7. *P. protegens* CHA0 forms tightly packed colonies in crevices between the rhizodermal cells. The roots were observed 14 days post-inoculation. *P. protegens* CHA0 is labeled with mNeonGreen (pSW002-Pc-mNeonGreen). The images depict representative slices through a 5.6 µM Z-stack displayed as confocal (A-C) and confocal/brightfield overlay images (D-F). Relative distances through the Z-stack: (A, D) 0 µM, (B, E) 3.6 uM, (C, F) 5.6 µM.

Supplementary References

Collis, C. M., Kim, M., Partridge, S. R., Hall, R. M., and Stokes, H. W. (2002). Characterization of the Class 3 Integron and the Site-Specific Recombination System It Determines Characterization of the Class 3 Integron and the Site-Specific Recombination System It Determines. *Society*. 184, 3017–3026.

Desai, S., Naik, D., and Cumming, J. R. (2014). The influence of phosphorus availability and Laccaria bicolor symbiosis on phosphate acquisition, antioxidant enzyme activity, and rhizospheric carbon flux in Populus tremuloides. *Mycorrhiza*. 24, 369–382.

Hayashi, K., Shiina, T., Ishii, N., Iwai, K., Ishizaki, Y., Morikawa, K., and Toyoshima, Y. (2003). A role of the -35 element in the initiation of transcription at psbA promoter in tobacco plastids. *Plant Cell Physiol.* 44, 334–341.

Kim, J., and Mullet, J. E. (1994). Ribosome-binding sites on chloroplast rbcL and psbA mRNAs and light-induced initiation of D1 translation. *Plant Mol. Biol.* 25, 437–448.
